# Global Absence and Targeting of Protective Immune States in Severe COVID-19.

**DOI:** 10.21203/rs.3.rs-97042/v1

**Published:** 2020-10-28

**Authors:** Alexis J. Combes, Tristan Courau, Nicholas F. Kuhn, Kenneth H. Hu, Arja Ray, William S. Chen, Simon J. Cleary, Nayvin W. Chew, Divyashree Kushnoor, Gabriella C. Reeder, Alan Shen, Jessica Tsui, Kamir J. Hiam-Galvez, Priscila Muñoz-Sandoval, Wandi S Zhu, David S. Lee, Yang Sun, Ran You, Mélia Magnen, Lauren Rodriguez, Aleksandra Leligdowicz, Colin R. Zamecnik, Rita P. Loudermilk, Michael R. Wilson, Chun J. Ye, Gabriela K. Fragiadakis, Mark R. Looney, Vincent Chan, Alyssa Ward, Sidney Carrillo, The UCSF COMET Consortium, Michael Matthay, David J. Erle, Prescott G. Woodruff, Charles Langelier, Kirsten Kangelaris, Carolyn M. Hendrickson, Carolyn Calfee, Arjun Arkal Rao, Matthew F. Krummel

**Affiliations:** 1 Department of Pathology, San Francisco, 513 Parnassus Ave, HSW512, San Francisco, CA 94143-0511, USA; 2 ImmunoX Initiative, San Francisco, 513 Parnassus Ave, HSW512, San Francisco, CA 94143-0511, USA; 3 UCSF CoLabs, San Francisco, 513 Parnassus Ave, HSW512, San Francisco, CA 94143-0511, USA; 4 Department of Radiation Oncology, San Francisco, 513 Parnassus Ave, HSW512, San Francisco, CA 94143-0511, USA; 5 Division of Pulmonary and Critical Care Medicine, Department of Medicine and the Cardiovascular Research Institute, San Francisco, 513 Parnassus Ave, HSW512, San Francisco, CA 94143-0511, USA; 6 Departments of Otolaryngology, San Francisco, 513 Parnassus Ave, HSW512, San Francisco, CA 94143-0511, USA; 7 Department of Microbiology & Immunology, San Francisco, 513 Parnassus Ave, HSW512, San Francisco, CA 94143-0511, USA; 8 Sandler Asthma Basic Research Center, San Francisco, 513 Parnassus Ave, HSW512, San Francisco, CA 94143-0511, USA; 9 Institute of Human Genetics and Division of Rheumatology, Department of Medicine, San Francisco, 513 Parnassus Ave, HSW512, San Francisco, CA 94143-0511, USA; 10 Weill Institute for Neurosciences, Department of Neurology, San Francisco, 513 Parnassus Ave, HSW512, San Francisco, CA 94143-0511, USA; 11 Division of Infectious Disease, Department of Medicine, San Francisco, 513 Parnassus Ave, HSW512, San Francisco, CA 94143-0511, USA; 12 Division of Hospital Medicine, San Francisco, 513 Parnassus Ave, HSW512, San Francisco, CA 94143-0511, USA

## Abstract

While SARS-CoV-2 infection has pleiotropic and systemic effects in some patients, many others experience milder symptoms. We sought a holistic understanding of the severe/mild distinction in COVID-19 pathology, and its origins. We performed a whole-blood preserving single-cell analysis protocol to integrate contributions from all major cell types including neutrophils, monocytes, platelets, lymphocytes and the contents of serum. Patients with mild COVID-19 disease display a coordinated pattern of interferon-stimulated gene (ISG) expression across every cell population and these cells are systemically absent in patients with severe disease. Severe COVID-19 patients also paradoxically produce very high anti-SARS-CoV-2 antibody titers and have lower viral load as compared to mild disease. Examination of the serum from severe patients demonstrates that they uniquely produce antibodies with multiple patterns of specificity against interferon-stimulated cells and that those antibodies functionally block the production of the mild disease-associated ISG-expressing cells. Overzealous and auto-directed antibody responses pit the immune system against itself in many COVID-19 patients and this defines targets for immunotherapies to allow immune systems to provide viral defense.

## One Sentence Summary

In severe COVID-19 patients, the immune system fails to generate cells that define mild disease; antibodies in their serum actively prevents the successful production of those cells.

**Figure 1:**
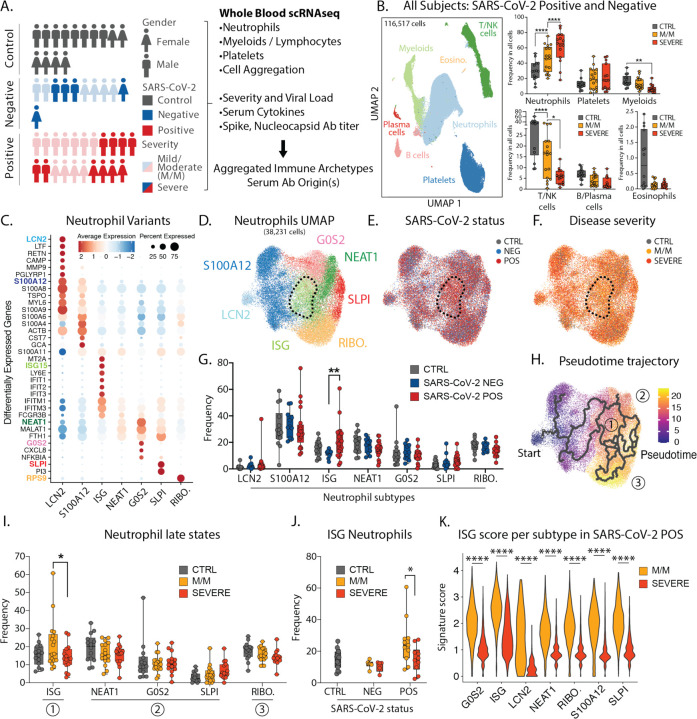
Severe COVID-19 disease is characterized by the lack of IFN-responsive neutrophils. **A**. Gender, SARS-CoV-2 status and disease severity in patients and control individuals (left) and description of study design (right). **B**. UMAP visualization of cells merged from the entire cohort with specific populations overlaid (left), and frequencies of these populations across control, mild/moderate (M/M) and severe individuals (right). **C**. Dotplot representation of top differentially-expressed-genes (DEG) between neutrophil subsets. **D**. UMAP visualization of neutrophil subsets. **E. and F**. Overlay of SARS-CoV-2 status and disease severity, respectively, on the neutrophil UMAP. **G**. Frequencies of neutrophil subsets among all neutrophils across control, SARS-CoV-2 negative and SARS-CoV-2 positive individuals. **H**. Pseudotime trajectory of neutrophil subsets. **I**. Frequencies of the neutrophil subsets among all neutrophils at later stages of pseudotime trajectories across control, mild/moderate and severe individuals. **J**. Frequency of ISG neutrophils among all neutrophils across SARS-CoV-2 status and disease severity. **K**. Score of ISG signature across neutrophil subtypes and disease severity in SARS-CoV-2 positive patients. Statistical significance was assessed using a two-way ANOVA test with multiple comparisons for panels B, G, I and J, and using a Wilcoxon test for panel K. * p-value < 0.05; ** p-value < 0.01; *** p-value < 0.001; **** p-value < 0.0001.

To understand immune biology amongst COVID-19 patients, we compared them to patients presenting with similar respiratory symptoms but who were not infected with the SARS-CoV-2 virus. We prospectively enrolled 21 SARS-CoV-2 positive inpatients, 11 inpatients with similar clinical presentations consistent with acute lung injury (ALI) or acute respiratory distress syndrome (ARDS), who were SARS-CoV-2 negative—those caused by other infections or of unknown origin—and 14 control individuals. We further categorized these over the next weeks as ‘mild/moderate’ (M/M: typically short stays in hospital with no need for mechanical ventilation and intensive care) or ‘severe’ (requiring intubation and intensive care) according to the full clinical course of their disease (Fig 1A/S1A and Table S1). Hence, our study includes patients with mild/moderate (n=11) or severe (n=10) COVID-19 and patients with mild/moderate (n=6) or severe (n=5) non-COVID-19 ALI/ARDS. With the exception of one individual, all our patients who presented with mild/moderate disease remained mild/moderate during hospitalization (Fig S1A), suggesting that mild/moderate and severe are more stable states rather than transient phases of disease in this cohort.

Since the majority of COVID-19 mortality is among patients with the (ARDS)—characterized by an exuberant immune response with prominent contributions from neutrophils, monocytes, platelets—we focused upon faithfully collecting these cells along with other major populations. We thus processed early morning blood samples from all individuals within 3 hours of sampling, and after red blood cell lysis, we analyzed the remaining white blood cells by single-cell RNA sequencing (scRNA-seq). After merging, batch-correction and doublet-removal our data comprised 116,517 cells (Fig 1B/S1B) among which we identified neutrophils, platelets, mononuclear phagocytes, T/NK cells, B cells, plasma cells and eosinophils (Fig S1C). We confirmed a positive association between neutrophil count and disease severity and an inverse correlation for lymphoid populations (Fig 1B/S1D) (1–3). At this level of resolution, findings were similar between SARS-CoV-2 negative and positive individuals (Fig S1E).

Within the neutrophils, we identified seven subtypes (Fig 1C/D), consistent with previous studies (2, 4). One population, harboring a strong interferon-stimulated gene (ISG) signature and henceforth termed ISG neutrophils, was highly enriched in SARS-CoV-2 positive patients but not in those whose disease was severe (Fig 1E/F/G). Analysis of populations using a pseudotime method to estimate differentiation trajectories (5) assigned the starting population as the stem LCN2 population (Fig 1H/S1F–G) and suggested three putative late populations: the ISG-expressing population (state 1), a collection of populations sharing expression of NEAT1, MALAT1 and FTH1 (state 2), and a population enriched for ribosomal genes (RIBO.; state 3) which may be en route to cell death. Of these late stages, the ISG subtype was the only one found significantly altered between mild/moderate and severe patients (Fig 1I) and specifically within the SARS-CoV-2 positive individuals (Fig 1J/S1H). ISG signature genes include master anti-viral regulators such as ISG15 and IFITM3 which restricts viral entry into the cytosol (6).

We also undertook a second form of analysis of differentially expressed genes (DEG) from SARS-CoV-2 positive versus negative patients, and from mild/moderate versus severe patients across all neutrophils (Fig S1I–L). This demonstrated that ISG signature genes are differentially higher in all neutrophils, of all subsets, specifically in SARS-CoV-2 positive mild/moderate patients, than in SARS-CoV-2 positive patients with severe disease (Fig 1K/S1M–O). In contrast, a separate neutrophil degranulation gene program is upregulated in mild/moderate as compared to severe disease regardless of COVID status (Fig S1P–Q). This suggests a shared program of degranulation enhancement in all respiratory infections regardless of causative pathogen, and a global rise of the ISG program in all neutrophils in mild/moderate SARS-CoV-2 positive cases that is absent in severe SARS-CoV-2 infection (3).

**Figure 2:**
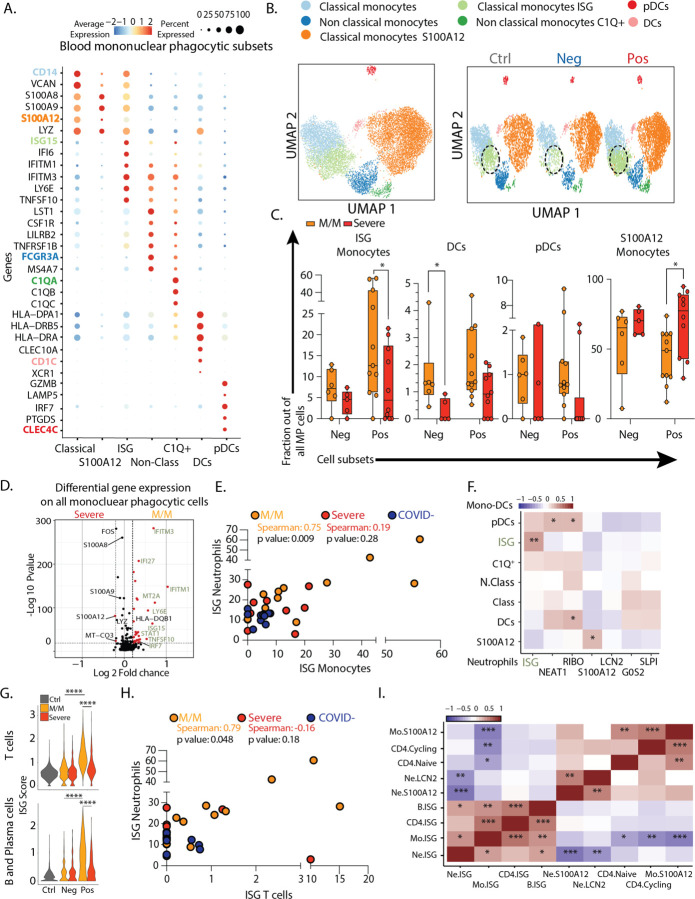
Severe COVID-19 disease is defined by the lack of a concerted IFN-response across peripheral blood immune cells. **A**. Dotplot representation of the top differentially-expressed-genes (DEG) between clusters identified in blood mononuclear phagocytic cell (MPC) subsets. **B**. UMAP visualization of the 19,289 MPC isolated from the entire dataset (left) and splitted by SARS-CoV-2 status (right). **C**. Frequencies of MPC subsets among all MPC across control, mild/moderate (M/M) and severe individuals **D**. Volcano plot showing results of differential gene expression (DGE) analysis performed on all MPC between mild/moderate (right) and severe (left) patients. **E**. Scatter plot between neutrophil and monocyte ISG positive subsets patient by patient. **F**. Correlation matrix using Spearman Rank Correlation between the frequency of all neutrophils and monocytes subtypes in all SARS-CoV-2 negative and SARS-CoV-2 positive patients. (n=32) **G**. Violin plot of ISG signature on all T cells (top) and all B/Plasma cells (bottom) across SARS-CoV-2 status and disease severity. **H**. Scatter plot between neutrophil and CD4 T cell ISG positive subsets patient by patient. **I**. Correlation matrix using Spearman Rank Correlation between the most and the least correlated cell subsets to the Neutrophils ISG positive cells (data include all SARS-CoV-2 negative and positive patients). Statistical significance was assessed using Spearman method (n=32) (G.) Kruskal Wallis test with multiple comparisons (C.). * p-value < 0.05; ** p-value < 0.01; *** p-value < 0.001; **** p-value < 0.0001.

Assessing the mononuclear phagocytes—monocytes, macrophages, dendritic cells and plasmacytoid dendritic cells (pDC)—yielded 7 clusters of transcriptionally distinct cells subsets, evenly distributed across our cohort with a heterogenous number of genes and unique molecular identifiers detected for each cluster (Fig 2A–B/2SA–D). We identified ISG classical monocytes as being enriched in SARS-CoV-2 positive patients, and particularly those having mild/moderate disease, similarly to neutrophils (Fig 2B–C/S2E–G). pDCs which are substantial producers of the cytokine IFNα are also typically elevated in mild/moderate SARS-CoV-2 positive patients although this falls short of statistical significance in our dataset (Fig 2C). In contrast, elevated DCs that were previously considered a hallmark of COVID-19 patients when compared to healthy controls (2) are also elevated in SARS-CoV-2 negative patients (Fig 2C). ISG monocytes also expressed genes associated with glycolysis, compared to the S100A12 subset that were enriched for genes associated with OxPhos metabolism, consistent with previous reports in bacterial sepsis (7) (Fig S3A). As for neutrophils, differential gene expression analysis demonstrated that ISGs were the dominant genes associated with mild/moderate phenotypes when the entire mononuclear phagocyte pool was assessed (Fig 2D).

ISG monocyte and ISG neutrophil frequencies were strongly correlated with one another in mild/moderate SARS-CoV-2 positive individuals (Fig 2E). Correlating multiple neutrophil subsets versus mononuclear phagocyte subsets across the entire cohort confirmed the strong correlation between ISG neutrophils and ISG monocytes and highlighted a significant correlation between pDCs and NEAT1 neutrophils, which have been previously described in viral infection (30072977) (Fig 2F). Similarly, we performed a comprehensive analysis of T cell and B cell frequencies (Fig S4) and found again that both cell types are significantly enriched in ISG signatures, specifically in mild/moderate COVID-19 patients (Fig 2G). On a patient-by-patient basis, the populations of ISG+ in one compartment correlated with the frequency of ISG expressing cells in another, for example of ISG+ T cells and ISG+ neutrophils, uniquely in mild/moderate patients (Fig 2H). Spearman correlation analysis across multiple cell types in all patients thus showed a collection of correlated ISG populations and a second anti-correlated block of other cell populations, notably those expressing S100A12 (Fig 2I).

**Figure 3:**
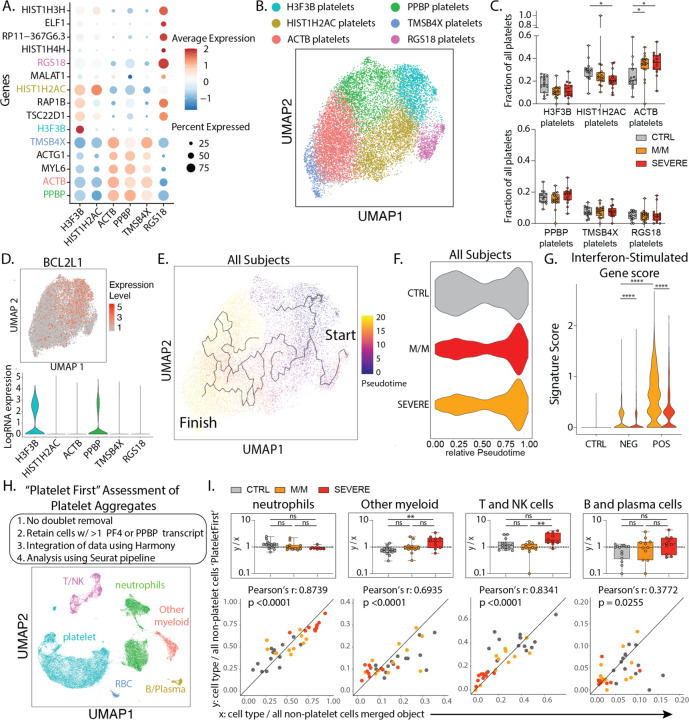
Platelet subtypes and putative platelet aggregates in COVID-19 disease. **A**. Dotplot representation of the top DEG between clusters identified in the platelet subset. **B**. UMAP visualization of 16,903 platelets isolated from the entire dataset showing various subsets, colored distinctly by their identity. **C**. Frequencies of the identified clusters among all platelets in healthy donors and all patients with mild/moderate (M/M) and severe disease. **D**. UMAP visualization of all platelets colored by *BCL2L1* (top) and violin plot of *BCL2L1* expression level across all identified platelet subsets. **E**. UMAP visualization of all platelets with overlay of Pseudotime trajectory. **F**. Violin plot of the relative Pseudotime of all platelets split by healthy donors, mild/moderate and severe patients. **G**. ISG signature score in all platelets across SARS-CoV-2 status and disease severity. **H**. Outline of ‘Platelet First’ assessment to identify platelet aggregates in entire whole blood scRNA-seq data set. UMAP visualization of the 52,757 putative platelet aggregates with specific populations overlaid. **I**. Bottom: Scatter plot of cell type frequency within merged object of entire cohort shown in Figure 1B (*x*-axis) versus same cell type frequency within ‘Platelet First’ object (*y*-axis). The identity line *x=y* is drawn as a reference. Each dot represents a healthy control or SARS-CoV-2 positive patient sample and are color-coded by disease severity. Pearson r correlation coefficient and two-tailed p value are shown for each cell type. Top: Box plots of *y/x-*ratio for each healthy control or patient sample, separated by disease severity. Differences in C. and I. were calculated using a two-way ANOVA test with multiple comparisons. * p.value < 0.05; ** p.value < 0.01; *** p.value <0.001; **** p.value < 0.0001; ns: non-significant.

Platelets are the mediators of blood coagulation and their activation can be associated with inflammation and infectious diseases, termed immunothrombosis(8). In patients infected with SARS-CoV-2, 25–33% of patients present with thrombocytopenia or thromboembolic events(9). We sought to determine how this might be reflected in changes in the expressed genes and concomitantly in the heterotypic cell doublet frequencies in SARS-CoV-2 infections versus other respiratory infections. Our scRNA-seq whole blood data set allowed us to identify and subset platelets based on established platelet signature genes: PPBP, PF4, CLEC1B, RGS10, RGS18 (Fig 1B/S1C). Analysis of these platelets revealed six clusters (Fig 3A/B), including three subsets (“H3F3B”, “HIST1H2AC”, and “RGS18”) characterized by high expression levels of histone protein-encoding transcripts. Such populations may represent early platelets, based upon the suggestion that these anucleated cells carry transcripts acquired from their parental cells, megakaryocytes, during recent platelet formation(10). One by one comparison of these platelet subtypes among healthy controls and patients with mild/moderate and severe disease revealed only minor depletion of HIST1H2AC platelets with disease severity and increase in ACTB platelets—expressing genes associated with cytoskeletal functions—in both mild/moderate and severe patients (Fig 3C).

To compare platelet turnover between disease severity groups, we overlaid the expression of BCL2L1 onto our platelet data set. This transcript encodes the anti-apoptotic protein Bcl-xL, which has been identified as a ‘molecular clock’ for platelet lifetime(11). This identified the H3F3B cluster as representing ‘young’ platelets (Fig 3D), a result supported by a second signature of transcripts in young, reticulated platelets(12) (Fig S5A). This population was thus supported as the starting point for a pseudotime analysis, in which the histone-high clusters (H3F3B, HISTH2AC, RGS18) and cytoskeletal protein-high clusters (ACTB, PPBP, TMSB3X) were observed at the start and end of the pseudotime trajectory, respectively (Fig 3E/S5B). Plotting the platelet cell frequencies of our cohort along the trajectory suggested that platelets from all patients with disease were broadly overrepresented at the end of the trajectory (Fig 3F), supporting a discrete and systemic relative loss of young platelets in all patients as compared to controls. Platelets did not harbor an ISG-specific cell cluster (Fig S5C), but akin to myeloid and lymphoid cells, the ISG score in all platelets from mild/moderate patients was increased relative to controls and was comparatively decreased in severe patients, particularly in SARS-CoV-2 infected individuals (Fig 3G).

Taking advantage of this unique dataset, we explored the identification of heterotypic aggregates between platelets and non-platelets by using a ‘Platelet First’ approach (Fig 3H, see methods). This approach analytically prioritized capture of every cell that was aggregated with a platelet, prior to doublet assessment. The ‘Platelet First’ object revealed the presence of platelet transcripts associated with cells that also bore signatures of other major blood cell types (Fig 3H/S5D–E). We separately analyzed a smaller object that included only libraries containing samples pooled prior to cell encapsulation, allowing us to assess inter-patient doublets to conclude that the majority of these aggregates were not formed during the scSeq pipeline (Fig S5F). In plotting the blood cell frequencies within the ‘Platelet First’ object against the cell frequencies within the original data set, we found a largely linear relationship between the frequency of a given aggregating population (x-axis) and the frequency with which that cell is found in an aggregate (y-axis) (Fig 3I). This first-order linear relationship suggests that, at least in circulating blood, platelets form aggregates indiscriminately with varying other cell types without favoring one or the other. Possibly activated vasculature would provide a cue that would change this pattern of aggregation.

**Figure 4:**
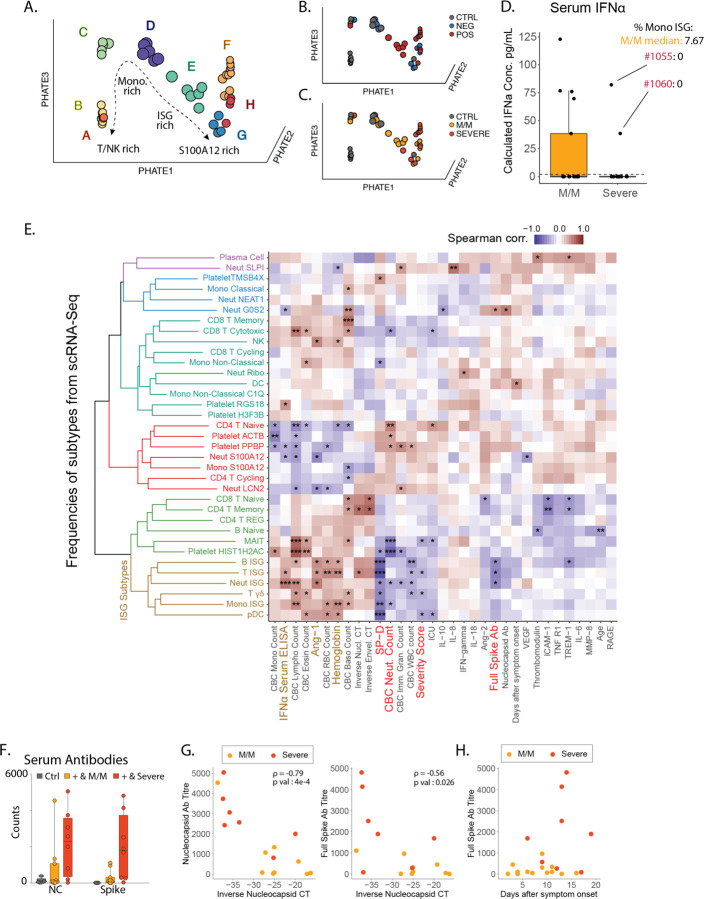
Integrated view of Blood Composition in COVID-19 Patients. **A-B**. 3D PhEMD embedding of all patients, colored by **A**. de novo patient clusters A-H, **B**. SARS-CoV-2 status, and **C**. disease severity. **D**. Measurement of serum IFNα concentration from SARS-CoV-2 positive and negative patients by ELISA. Patients 1055 and 1060 are highlighted in red and their Monocytes ISG frequency from Fig 2C is noted as well as the median for mild COVID-19 mild/moderate patients. **E**. Matrix of Spearman correlation coefficients between all subtype frequencies (out of major cell types, e.g. Neut ISG out of all Neutrophils) obtained from scRNA-Seq versus patient metadata, viral load, Ab titers, and serum analyte levels on a patient-by-patient basis excluding healthy controls. Patients for which data were unavailable were excluded from correlation analysis for each comparison. Variables on both axes were ordered via hierarchical clustering with the computed dendrogram displayed for subtype frequencies. This dendrogram was divided into 6 groupings with the one containing ISG+ subtypes highlighted in brown. Clinical variables generally correlated with severity highlighted in red and anti-correlated in brown. (n for correlation comparisons ranged from n=14 to 32) * p<0.05, ** p<0.005, *** p<0.0005. **F**. Measurement of anti-SARS-CoV-2 antibody levels in serum from patients by Luminex assay (M/M: Mild/Moderate). **G**. Scatter plots showing viral load versus levels of antibody binding SARS-CoV-2 Nucleocapsid and Full Spike protein for patients in the cohort with severity overlaid. Antibody levels are shown as arbitrary units of MFI from Luminex assay while viral load is represented by an inverse CT number from QRT-PCR with target amplification of the SARS-CoV2 Nucleocapsid sequence. Correlation coefficient and significance calculated using Spearman’s method. Patients for which data was unavailable were excluded. (n=16) **H**. Scatterplot for SARS-CoV2 Full Spike protein antibody titers relative to days post symptom onset. Patients for which data was unavailable were excluded. (n=22)

After observing that ISG expression profiles were elevated in every cell type among pateints with mild/moderate disease and COVID-19 but globally reduced with severe COVID-19 disease, we turned our attention to a hypothesis generating holistic approach to data analysis. In an attempt to visualize the global shift in gene expression data across cell types to identify trends with clinical correlates. We first undertook a phenotypic earth mover’s distance (PhEMD) approach (13) that identifies and differentiates joint cell frequency patterns to highlight sources of patient-to-patient variation. PhEMD embedding of patients based on their subtype frequencies revealed eight distinct groups of patients (Fig 4A/S6A) where progression from A through H represent patients with generally increasing relative frequency of neutrophils, C, D, G and H include patients with relative enrichment in monocytes and E represents patients with an enrichment of ISG neutrophils and mostly consists of SARS-CoV-2 positive patients with mild/moderate disease (Fig 4B–C). In contrast, Group G, which is an alternative and ‘severe’ fate for patients is enriched for neutrophil and has a high ratio of cell frequency of S100A12 to ISG neutrophils (Fig S6A).

When we examined serum IFNα levels, we found that mild/moderate individuals made more of this cytokine on average as compared to severe patients, which would be consistent with higher levels of ISG cell populations, however there were patient with severe disease individuals who also made high levels of IFNα (Fig 4D). To integrate the scRNA-Seq cell populations in the context of other clinical and serum fractions, we constructed a Spearman correlation matrix comparing all subtype frequencies described above with a collection of serum cytokines, antibodies and clinical variables (Fig 4E). Following hierarchical clustering of variables, ISG subtypes cluster together and are correlated with serum IFNα concentrations, consistent with these cells arising in response to globally high concentrations of this cytokine as shown previously (3) (Fig S6B). ISG-expressing populations are also associated with low severity of COVID-19 illness, lower plasma levels of SP-D (indicative of alveolar epithelial injury) and, only modestly, with IL-6 levels. We also note the absence of significant correlation between ISG populations and days after symptoms onset, indicative of a disease ‘state’ (Fig 4E and Fig S1A). We further correlated our subtype frequencies against a high-dimensional panel of plasma protein levels (Fig S6B) and again observed clustering of most ISG subtypes, which correlated with a host of factors indicative of a strong ISG and Th1 response (CXCL1/6/10/11, TNFB, IL-12B, MCP-2/4), while inversely correlated with others (CCL23, MMP10, HGF). An unexpected anticorrelate of the ISG state was the concentration of serum antibodies against the SARS-CoV-2 Spike and Nucleocapsid proteins.

This anticorrelation was profound (Fig 4F) and we considered it a paradox that severe patients have higher levels of potentially neutralizing antibodies. This is in apparent contradiction with a previous report showing that viral load is associated with severity and mortality in COVID-19(14, 15) a difference which could be explained by the fact that these studies focus on patients with high mortality, which was a very rare event in our cohort (Sup Table S1). Both antibody specificities were anticorrelated with the viral load as assessed from nasal swabs (Fig 4G) consistent with though not definitive for being neutralizing. As increased antibody titers and decreased viral load have been reported to be a feature of later disease stage(16), we considered the hypothesis that mild/moderate disease – characterized by high frequency of ISG+ signatures – would simply precede severe disease states. However, antibody titers in severe patients are consistently higher compared to mild/moderate patients even two weeks beyond symptom onset (Fig 4H), and only one of our 11 mild/moderate patients would go on to exhibit a severe disease (Fig S1A). Moreover, we observed no statistical correlation between days of onset and the presence of ISG+ cell populations (Fig 4E). These elements would seem to argue against a simple time relationship between mild/moderate and severe states.

**Figure 5:**
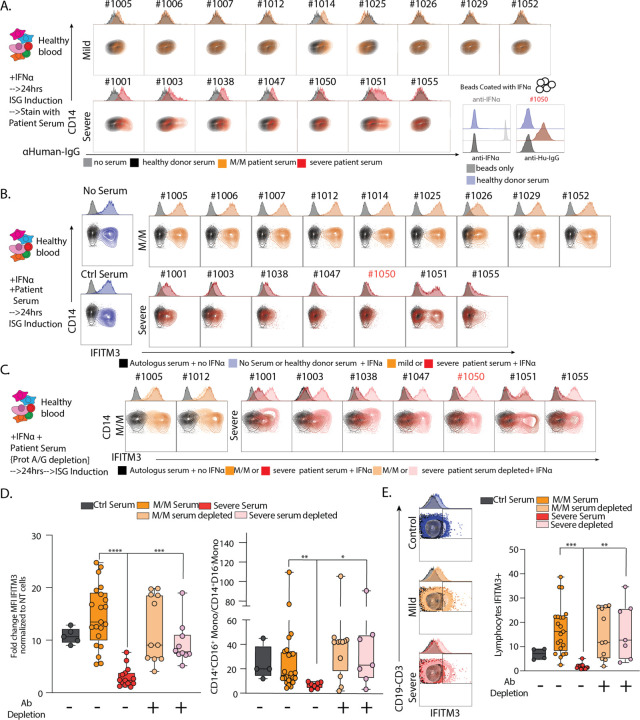
Neutralization of ISG induction by Antibodies from Severe COVID-19 Patients. **A**. Contour plots and histograms of CD14 Monocytes from healthy blood cultured with IFNα to induce expression of ISGs and stained with serum from heathy donor, mild/moderate (M/M) or severe SARS-CoV-2 positive patients with secondary staining with α-human IgG. Bottom right: histogram of beads coated with IFNα and stained with an antibody raised against IFNα or serum from severe SARS-CoV-2 positive patient #1050 or healthy donor. Black histograms represent non coated beads. **B-C**. Contour plots and histograms of CD14 Monocytes from healthy blood cultured with IFNα and serum from heathy donor, mild/moderate or severe SARS-CoV-2 positive patient quantifying levels of intracellular IFITM3 staining. **C**. Mild/Moderate (light yellow) or Severe (pink) sera were pre-treated with protein G/A before incubation with PBMC. **D**. Box plot of IFITM3 induction in CD14 monocytes (left) and intermediate to classical monocytes ratio (right) from 2 different experiment and 2 different healthy donors. **E**. Left: Contour plots and histograms of pooled CD3+/CD19+ lymphocytes from healthy blood cultured with IFNα and serum from heathy donor, mild/moderate or severe SARS-CoV-2 positive patients. Mild/moderate (light yellow) or Severe (pink) sera were pre-treated with protein G/A before incubation with PBMC to deplete antibodies. Right: Box plot of IFITM3 induction in lymphocytes. Differences in D. and E. were calculated using a two-way ANOVA test with multiple comparisons. * p.value < 0.05; ** p.value < 0.01; *** p.value <0.001; **** p.value < 0.0001; ns: non-significant.

Returning to the profound antibody reactivity and the global loss of ISG populations even in the presence of serum IFNα (Fig 4D), we hypothesized that phenotypic differences in our two groups of COVID-19 patients might also be mirrored or influenced or by systemic factors such as those carried in the blood and affecting all cell populations. We thus first asked whether serum from severe patients contained antibodies against ISG-expressing cells by directly applying serum to healthy peripheral blood mononuclear cells (PBMCs) from heathy individuals that were first induced to express IFITM3 (part of our ISG signature, encoding a protein that blocks viral entry (6)) by culturing them with IFNα for 24 hours, followed by flow cytometry analysis of monocytes and lymphocytes (Fig S7A). While we observed only low levels of serum IgG binding in serum from 1/9 mild/moderate patients, sera from 5/7 patients with severe COVID-19 illness displayed significant binding (Fig 5A/S7B). Staining was more pronounced on monocytes as compared to T and B cells which were largely stained with similar frequencies when using mild/moderate or severe serum (Fig S7C). When examining specificities in those patients that did not stain ISG-differentiated cells directly, we found that serum from patient 1050 produced antibodies that were specific for IFNα (**right inset** Fig5A), consistent with a very recent study (17) that also found these in approximately 12% of COVID patients. This finding nevertheless does not explain patients lacking ISG cells despite presence of IFNα in their serum (Fig 4D).

We then asked whether factors in the serum of severe patients affect the induction of the ISG signature gene pattern, including IFITM3, in response to culture with IFNα. We thus added patient serum at 10% into the IFNα stimulation conditions and found that, whereas control serum or serum from mild/moderate patients had no effect on differentiation as measured by either IFITM3 level or the frequencies of CD14+CD16^+^ intermediate monocytes produced (Fig 5B/5D and Fig S7D), all severe patient serum tested had profound effects on differentiation, varying from absolute block to partial inhibition.

To test whether these effects on IFN-induced production of ISG cell populations were in fact mediated by antibodies, we pre-adsorbed patients’ sera with Protein A/G beads to deplete them. This antibody depletion restored both IFITM3 induction and the total yield of interferon-stimulated monocyte cells (Fig 5C/D S7D). A similar block of ISG signature generation in response to IFNα was observed for other populations including lymphocytes, showing that this effect was global and similarly mediated by antibodies in serum severe patient (Fig 5E/S7D–E).

Taken together, this shows that an antibody response in severe patients targets ISG cell populations and their generation. In our cohort, this general effect of antibody manifests in all severe patients, whereas antibodies against the cytokine IFNα itself were seen only in one of seven patients, a similar frequency recently reported(17). Antibodies in many of these patients have direct specificity for determinants on the surface of ISG monocytes. The molecular specificities of these other antibodies are likely to be many and varied based on the differing patterns in this relatively small sample set and it will remain to be determined how and why tolerance is broken to the ISG pathway, in the course of infection. One likely candidate for modulation of B cell response is direct infection of monocytes by SARS-CoV-2. *In vitro* incubation of the virus with healthy cells indeed results in intracellular expression of both IFITM3 (indicating activation of this program) and spike protein (Fig S7F). If, in an early immune response, the ISG program is preferentially presented alongside the proteins from the pathogen, and the immune system of the patient is not already self-tolerant to those ISG proteins due perhaps to a lifelong absence of their profound expression, tolerance to those cells and those proteins may be broken. Conversely, as inflammatory monocytes normally restrict antibody generation (18), their infection and lysis by virus may in turn release overexuberant B cell responses to many antigens, not just those that are newly produced during the infection. Regardless, this work suggests that targeting overexuberant and autoreactive B cells with drugs such as rituximab (19) or through introduction of IVIG(20), perhaps alongside introduction of convalescent serum-derived antibodies to provide ongoing viral protection, may be an avenue to defeat the global suppression of protective ISG mediated viral immunity.

## Supplementary Material

1
