# Supplementary material for: Global Absence and Targeting of Protective Immune States in Severe COVID-19
Source: Res Sq. 2020 Oct 28:rs.3.rs-97042. Preprint. [Version 1] doi: 10.21203/rs.3.rs-97042/v1 (PMC7605560; doi:10.21203/rs.3.rs-97042/v1)
Supplement: 1 [file NIHPPRS97042V1-supplement-1.pdf]

**Supplementary Materials For:**

**Title: Global Absence and Targeting of Protective Immune States in Severe COVID-19.**

Alexis J. Combes[1,2,3,‡,*], Tristan Courau[1,2,3,‡], Nicholas F. Kuhn[1,2,‡], Kenneth H. Hu[1,2,‡], Arja Ray[1,2,‡], William S. Chen[2,4], Simon J. Cleary[2,5], Nayvin W. Chew[1,2,3], Divyashree Kushnoor[1,2,3], Gabriella C. Reeder[1,2,3], Alan Shen[1,2,3], Jessica Tsui[1,2,3], Kamir J. Hiam-Galvez[2,6,7], Priscila Muñoz-Sandoval[2,7,8], Wandi S Zhu[2,7,8], David S. Lee[2,9], Yang Sun[2,9], Ran You[1,2], Mélia Magnen[2,5], Lauren Rodriguez[2,6], Aleksandra Leligdowicz[5], Colin R. Zamecnik[2,10], Rita P. Loudermilk[2,10], Michael R. Wilson[2,10], Chun J. Ye[2,9], Gabriela K. Fragiadakis[2,3,9], Mark R. Looney[2,5], Vincent Chan[1,2], Alyssa Ward[9], Sidney Carrillo[5], The UCSF COMET Consortium, Michael Matthay[5], David J. Erle[2,3,5], Prescott G. Woodruff[2,5], Charles Langelier[11], Kirsten Kangelaris[12], Carolyn M. Hendrickson[5], Carolyn Calfee[5], Arjun Arkal Rao[1,2,3,*] and Matthew F. Krummel[1,2,*]

---

**This PDF file includes:**

Supplemental Table S1
Supplemental Figures S1-S7
Materials and Methods and Associated References
Table of Authors from the UCSF COMET Consortium

## Supplemental Table 1

| COMET ID | SARS-CoV-2 Status | Disease Severity | Age | Gender (1=MALE, 2 =F) | Days between symptoms and sampling (* = asymptomatic) | Other Infection(s) | ICU during hospital stay | Days under mechanical Ventilation | Discharged |
|---|---|---|---|---|---|---|---|---|---|
| 1008 | NEG | Mild/Moderate | 35.7 | 1 | 20 | Bacterial | No | 0 | Yes |
| 1016 | NEG | Mild/Moderate | 63.5 | 2 | 4 | None | No | 0 | Yes |
| 1017 | NEG | Mild/Moderate | 65.2 | 2 | 9 | None | No | 0 | Yes |
| 1045 | NEG | Mild/Moderate | 82.6 | 1 | NA | None | Yes | 0 | Yes |
| 1049 | NEG | Mild/Moderate | 68.8 | 2 | 1 | Bacterial | No | 0 | Yes |
| 1062 | NEG | Mild/Moderate | 75.8 | 2 | NA | Bacterial | No | 0 | Yes |
| **COVID Negative Mild** | **Average Data:** | | **65** | **1.7** | **9** | | | | |
| **n=6** | **SD:** | | **16** | | **8** | | | | |
| 1009 | NEG | Severe | 67 | 1 | NA | Bacterial | Yes | 5 | yes |
| 1010 | NEG | Severe | 50.7 | 2 | 9 | Bacterial | Yes | 5 | Yes |
| 1020 | NEG | Severe | 78.6 | 1 | 5 | Bacterial | Yes | 2 | Yes |
| 1021 | NEG | Severe | 77 | 2 | 2 | Bacterial | Yes | 5 | Yes |
| 1046 | NEG | Severe | 49.6 | 1 | 3 | Bacterial | Yes | 1 | Yes |
| **COVID Negative Severe** | **Average Data:** | | **65** | **1.4** | **5** | | | | |
| **n=5** | **SD:** | | **14** | | **3** | | | | |
| 1005 | POS | Mild/Moderate | 83.4 | 1 | 9 | None | No | 0 | Yes |
| 1006 | POS | Mild/Moderate | 71.9 | 2 | 16 | None | No | 0 | Yes |
| 1007 | POS | Mild/Moderate | 55.3 | 1 | 13 | Bacterial | No | 0 | Yes |
| 1012 | POS | Mild/Moderate | 41.1 | 1 | 3 | None | No | 0 | Yes |
| 1014 | POS | Mild/Moderate | 58.6 | 1 | 4 | None | Yes | 0 | Yes |
| 1025 | POS | Mild/Moderate | 73.2 | 1 | NA* | None | No | 0 | Yes |
| 1026 | POS | Mild/Moderate | 45.5 | 2 | 6 | None | No | 0 | Yes |
| 1029 | POS | Mild/Moderate | 85 | 2 | 2 | Viral | No | 0 | Yes |
| 1044 | POS | Mild/Moderate | 73.9 | 1 | NA* | None | No | 0 | Yes |
| 1052 | POS | Mild/Moderate | 37.9 | 2 | 4 | Viral | No | 0 | Yes |
| 1054 | POS | Mild/Moderate | 25.8 | 1 | NA* | None | No | 0 | Yes |
| **COVID Positive Mild** | **Average Data:** | | **59** | **1.4** | **7** | | | | |
| **n=11** | **SD:** | | **20** | | **5** | | | | |
| 1001 | POS | Severe | 34.8 | 2 | 12 | Viral | Yes | 10 | Yes |
| 1002 | POS | Severe | 79.6 | 1 | 4 | Bacterial | Yes | 7 | Yes |
| 1003 | POS | Severe | 43.6 | 2 | 13 | Viral | Yes | 5 | Yes |
| 1031 | POS | Severe | 44.2 | 1 | 20 | Viral | Yes | 28 | Yes |
| 1038 | POS | Severe | 61.4 | 2 | 14 | None | Yes | ND | Yes |
| 1047 | POS | Severe | 48.3 | 2 | 6 | Viral + Bacterial | Yes | 28 | Yes |
| 1050 | POS | Severe | 55 | 1 | 13 | Bacterial | Yes | 29 | Yes |
| 1051 | POS | Severe | 63.3 | 1 | 19 | Bacterial | Yes | 33 | Yes |
| 1055 | POS | Severe | 62.4 | 1 | 4 | Viral | Yes | 31 | Yes |
| 1060 | POS | Severe | 44.1 | 1 | 6 | Bacterial | Yes | 5 | Yes |
| **COVID Positive Severe** | **Average Data:** | | **54** | **1.4** | **11** | | | | |
| **n=10** | **SD:** | | **13** | | **6** | | | | |
| ICC_0001 | CTRL | CTRL | 39 | 1 | NA | NA | NA | NA | NA |
| ICC_0003 | CTRL | CTRL | 24 | 1 | NA | NA | NA | NA | NA |
| ICC_0666 | CTRL | CTRL | 45 | 2 | NA | NA | NA | NA | NA |
| ICC_1084 | CTRL | CTRL | 34 | 1 | NA | NA | NA | NA | NA |
| ICC_1117 | CTRL | CTRL | 41 | 1 | NA | NA | NA | NA | NA |
| ICC_1367 | CTRL | CTRL | 52 | 1 | NA | NA | NA | NA | NA |
| ICC_3231 | CTRL | CTRL | 28 | 2 | NA | NA | NA | NA | NA |
| ICC_3954 | CTRL | CTRL | 37 | 2 | NA | NA | NA | NA | NA |
| ICC_4096 | CTRL | CTRL | 31 | 1 | NA | NA | NA | NA | NA |
| ICC_4117 | CTRL | CTRL | 59 | 2 | NA | NA | NA | NA | NA |
| ICC_4319 | CTRL | CTRL | 30 | 1 | NA | NA | NA | NA | NA |
| ICC_4444 | CTRL | CTRL | 34 | 2 | NA | NA | NA | NA | NA |
| ICC_4799 | CTRL | CTRL | 40 | 2 | NA | NA | NA | NA | NA |
| ICC_4923 | CTRL | CTRL | 34 | 1 | NA | NA | NA | NA | NA |
| **Healthy Controls** | **Average Data:** | | **38** | **1.4** | **NA** | | | | |
| **n=14** | **SD:** | | **9** | | **NA** | | | | |

**Supplementary Table 1: COVID-19 Whole Blood Study Cohort.** Patients were enrolled as described in Material and Methods and blood was collected from 4:30AM rounds on the first day after admission. Healthy controls were from volunteers who fasted overnight and were collected between 6am and 9am. Data on individuals is shown along with average and standard deviations.

## Supplementary Figures S1-S5

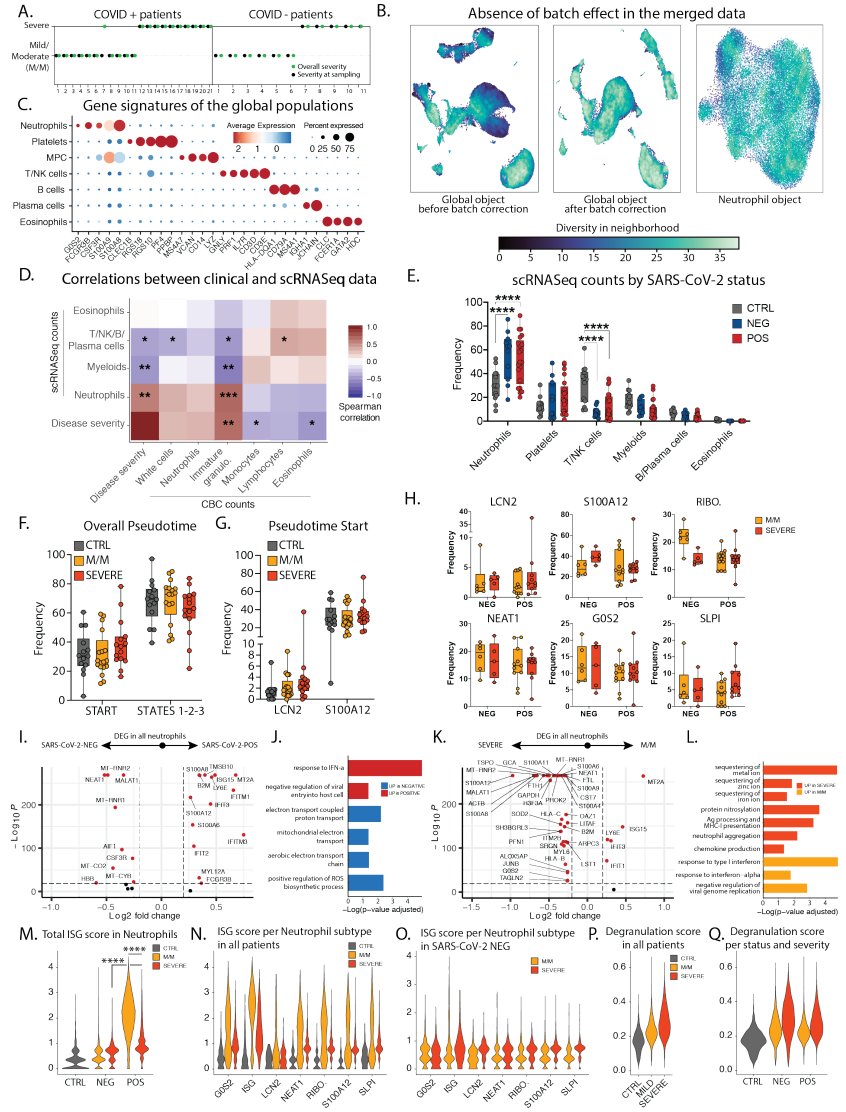


TANKA et al. Figure S1

**Supplementary Figure 1: A**. Patient symptoms plot: symptom at day of sampling (first day of admission to the hospital) is represented in black, while symptom based on the entire course of hospitalization is in green. In the rest of the manuscript we categorized patient into mild or severe cases based on all the entire course of hospitalization (green). **B**. Quantification of the batch effect using neighbor diversity score in the global object UMAP before (left) and after (middle) batch correction, along with the neutrophil (right) UMAP plot, as in Fig1B and Fig1D, using the diversity in neighborhood method. **C**. Dotplot representation of landmark genes expressed by global populations in Fig1B. **D**. Spearman's correlation comparison between between disease severity and population frequencies calculated from 10X scRNAseq analyses (10X) or complete blood cell counts (CBC). Patients for which CBC counts were unavailable were excluded. Significance was calculated using Spearman's method. * p value<0.05; ** p value <0.05; *** p value<0.005 (n=29) **E**. Frequency of the global populations in Fig1B among all cells across SARS-CoV-2 status. **F and G**. Frequencies of the neutrophil subsets among all neutrophils across control, mild/moderate (M/M) and severe individuals at the overall start/late states of the trajectories (F) or at specific early stages of the pseudotime (G). **H**. Frequency of the LCN2, S100A12, RIBO., NEAT1, G0S2 and SLPI neutrophils among all neutrophils across SARS-CoV-2 status and disease severity. **I to L**. Volcano plots showing DEG (I and K) and bar plots showing GO term enrichment from these DEG (J and L) between all neutrophils from either SARS-CoV-2 positive vs negative patients (I and J) or mild/moderate vs severe patients (K and L) **M to Q.** Scores of ISG signature (M to O) and neutrophil degranulation (P and Q) in either all neutrophils across control, mild/moderate an severe patients (P), all neutrophils across SARS-CoV-2 status and disease severity (M and Q) or specific neutrophil subtypes across severity in either all patients (N) or only SARS-CoV-2 negative patients (O). Statistical significance was assessed using a two-way ANOVA test with multiple comparisons for panels E, F and H, and using a Wilcoxon test for panel M. * p.value < 0.05; ** p.value < 0.01; *** p.value < 0.001; **** p.value < 0.0001.

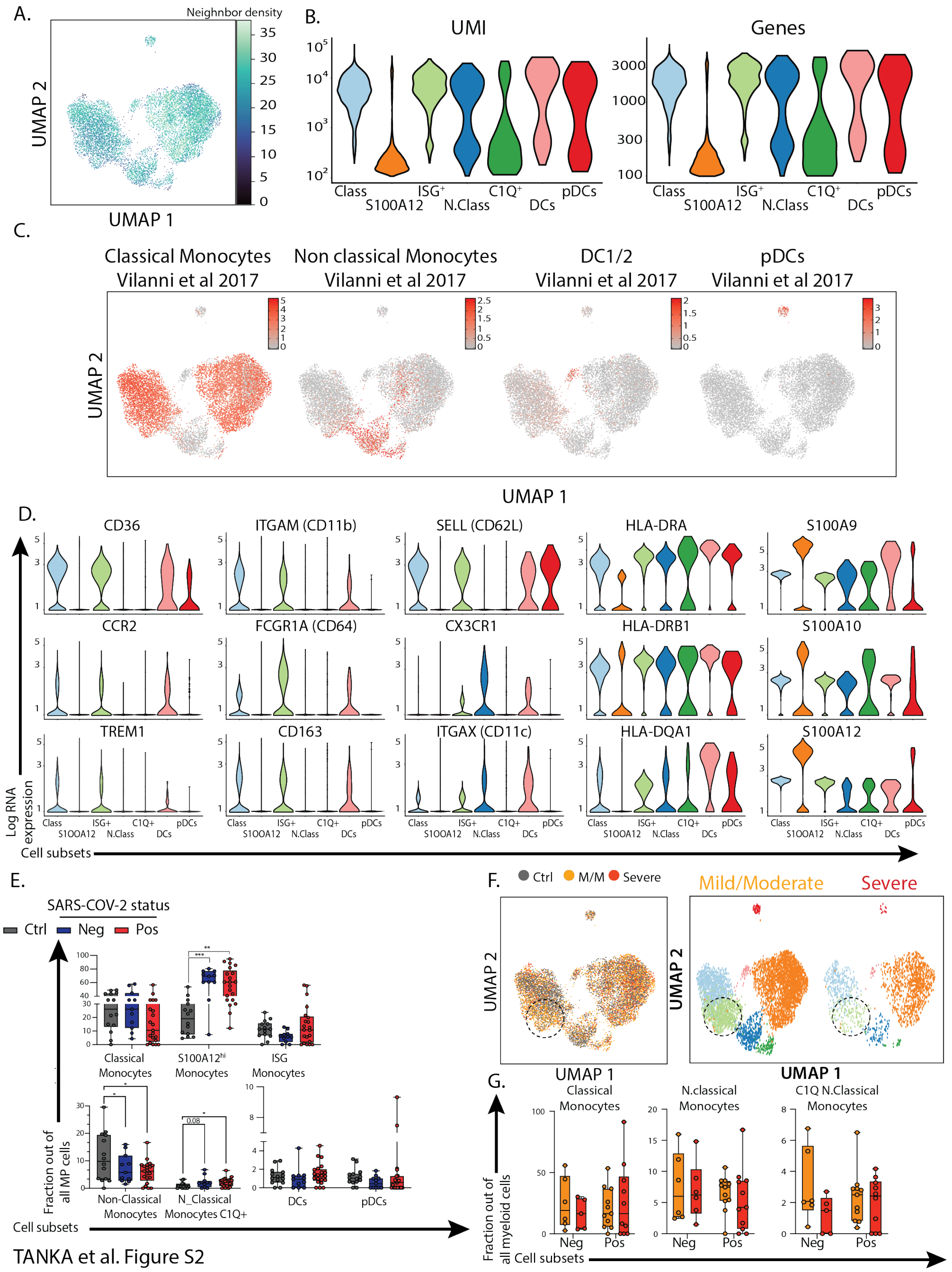


TANKA et al. Figure S2

**Supplementary Figure 2: A**. Quantification of the batch effect before and after batch correction using neighbor diversity score in the mononuclear phagocytic cells (MPC) object from UMAP plot in Fig2C, using the diversity in neighborhood method. **B**. Violin plot of number of unique genes (bottom) and number of unique molecules (top) detected from Single cell sequencing for each cluster identified in the MPC dataset. **C**. Overlay of previously described blood mononuclear phagocytic cell signature from healthy individual on MPC from UMAP plot in Fig2C. **D**. Violin plots of canonical genes previously described as expressed by blood MPC for each for each cluster identified in the MPC dataset. **E**. Frequencies of the MPC subsets among all MPC across control, SARS-CoV-2 negative and SARS-CoV-2 positive individuals. **F**. UMAP visualization of the 19,289 MPC colored (left) and split by (right) by disease severity. **G**. Frequencies of the classical monocytes, cycling monocytes, non-classical monocytes and C1Q+ non classical monocytes among all MPC across SARS-CoV-2 negative and SARS-CoV-2 positive individuals split it by disease severity. Statistical significance was assessed using a two-way ANOVA test with multiple comparisons. * p.value < 0.05; ** p.value < 0.01; *** p.value < 0.001; **** p.value < 0.0001.

A.

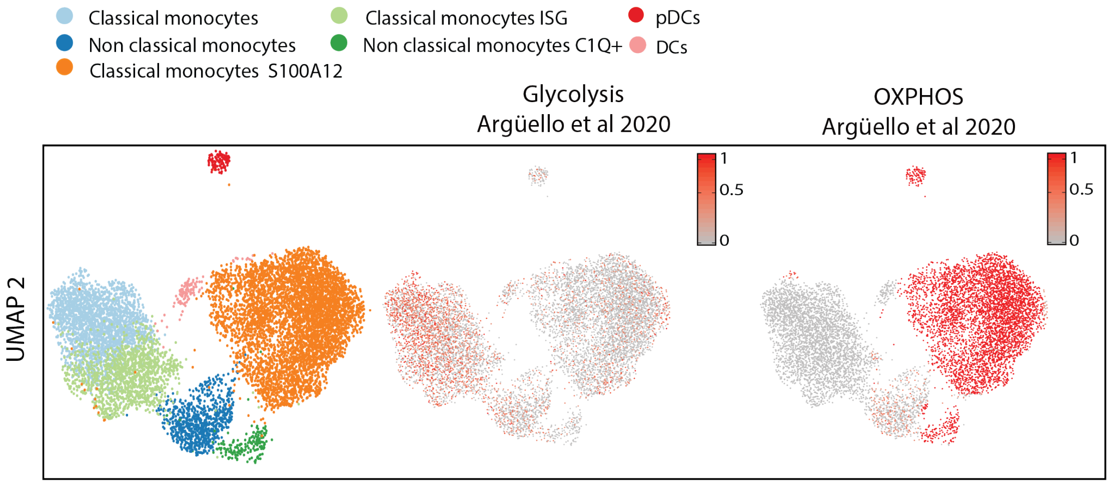


**Supplementary Figure 3. A.** Overlay of previously described glycolytic and oxidative phosphorylation gene signature on mononuclear phagocytic cells (MPC) from UMAP plot in Fig2C**.**

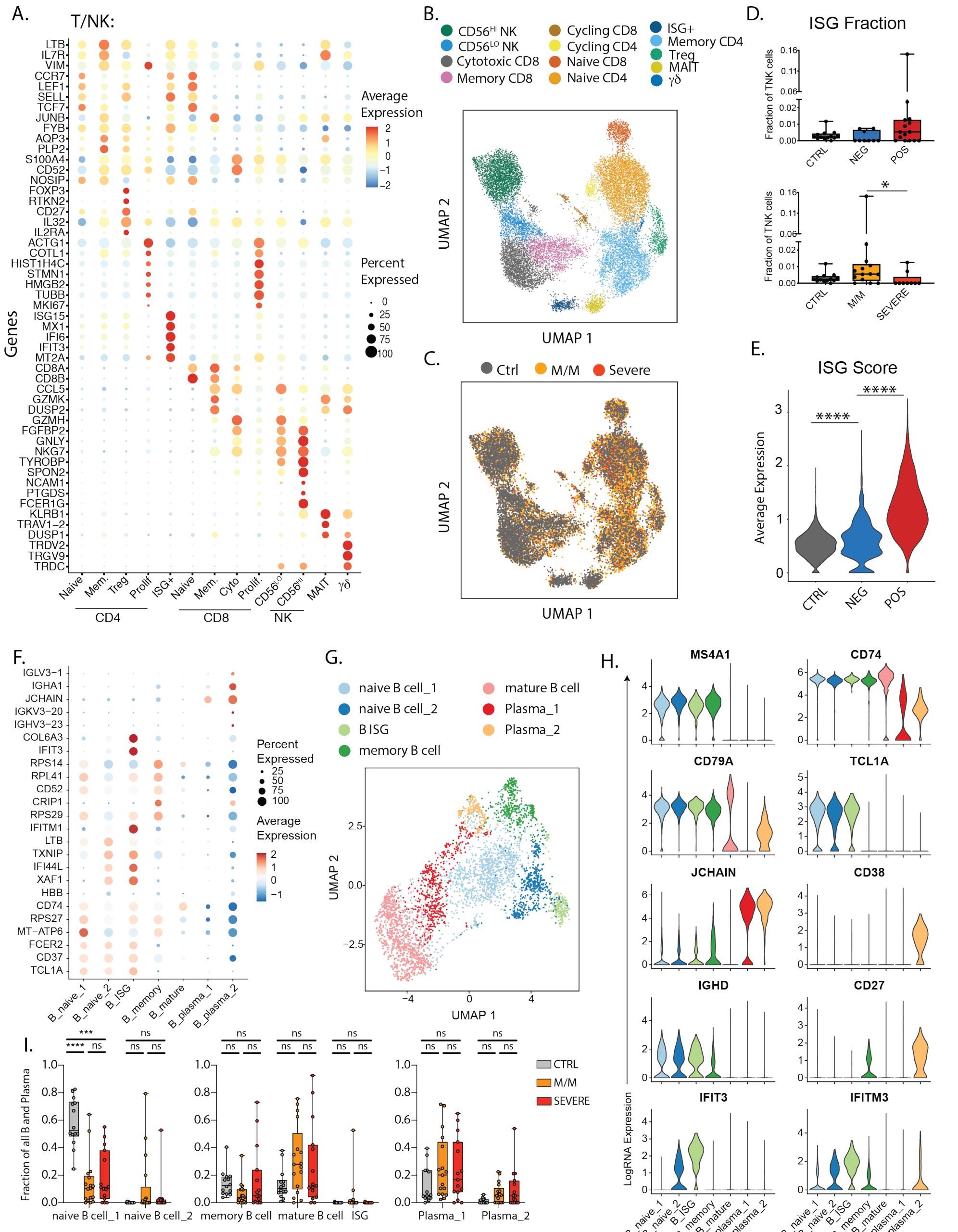

TANKA et al. Figure S4

**Supplementary Figure 4: A.** Dotplot representation of the top DEG between clusters identified in the T and NK cell subset. **B.** UMAP visualization of 16,708 T and NK cells in the entire dataset showing various subsets, colored distinctly by their identity. **C.** Overlay of the above UMAP of all T and NK cells, colored by disease severity underlining the lack of batch effects while merging the datasets from all patients. **D**. Abundance of the Interferon-stimulated-gene (ISG) + subset among all T and NK cells in healthy donors, SARS-CoV-2 negative and SARS-CoV-2 positive patients (top) and in healthy donors and patients with mild/moderate (M/M) and severe disease (bottom). **E.** ISG signature score between healthy controls, SARS-CoV-2 negative and SARS-CoV-2 positive patients. **F.** Dotplot representation of the top DEG between clusters identified in the B and plasma cell subset. **G.** UMAP visualization of 4,380 B and plasma cells isolated from the entire dataset showing various subsets, colored distinctly by their identity. **H.** Violin plots of canonical genes previously described as expressed by B and plasma cells for each identified cluster. **I.** Frequencies of the identified clusters among all B and plasma cells in healthy donors and patients with mild/moderate and severe disease. Differences in D. and E. were calculated using Kruskal-Wallis non-parametric ANOVA with multiple comparisons. * p <0.05 and **** p< 0.001. Differences in I. were calculated using a two-way ANOVA test with multiple comparisons. * p.value < 0.05 and **** p.value < 0.0001. ns, non-significant.

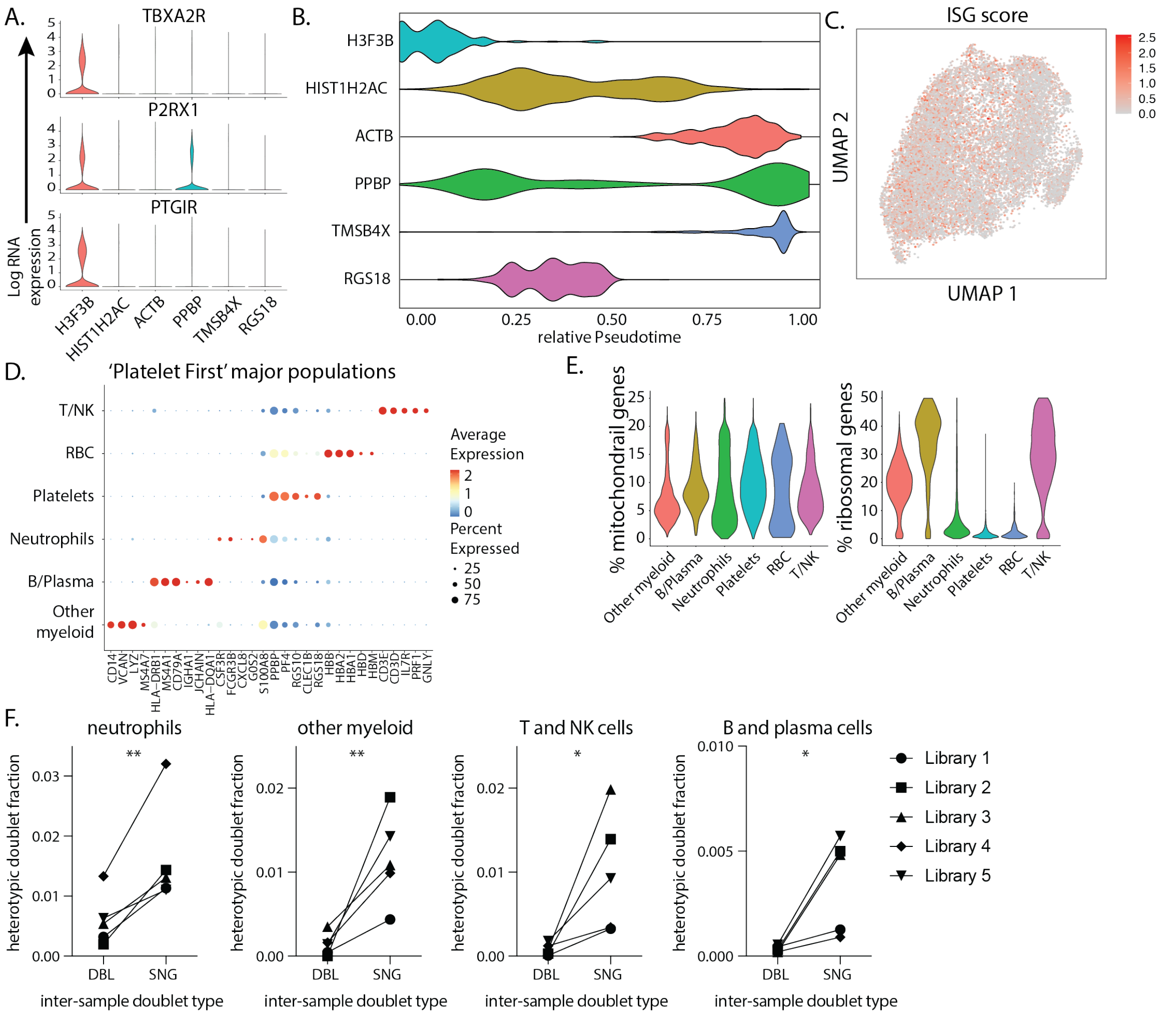


**Supplementary Figure 5: A.** Violin plots of genes identifying young, reticulated platelets (*1*) in the platelet dataset. **B.** Violin plots of the relative pseudotime of each platelet cell subset present in Figure 3B. **C.** UMAP visualization of all platelets colored by ISG score. **D.** Dotplot representation of the top DEG between clusters identified in the 'Platelet First' object. In this object (see also Fig 3) no doublet removal filtering step was applied to include all heterotypic cell-cell aggregates (Step 1). This was followed by retaining all cells with >1 platelet-specific transcripts PF4 or PPBP (Step 2). Step 2 guaranteed analysis of cell events and aggregates containing platelets. Identically to our original data set in Figure 1B, integration of data was done using Harmony (Step 3), and the 'Platelet First' object was then analyzed using the Seurat v3 pipeline (Step 4). **E.** Violin plots of the percentage of mitochondrial and ribosomal genes within clusters identified in the 'Platelet First' object. **F.** Inter-sample doublet rates in inferred platelet-involved heterotypic doublets show that platelet aggregates occur in vivo. DBL, doublet. SNG, singlet. Differences in F. were calculated using a one-sided Student's t test * p.value < 0.05 and ** p.value < 0.01.

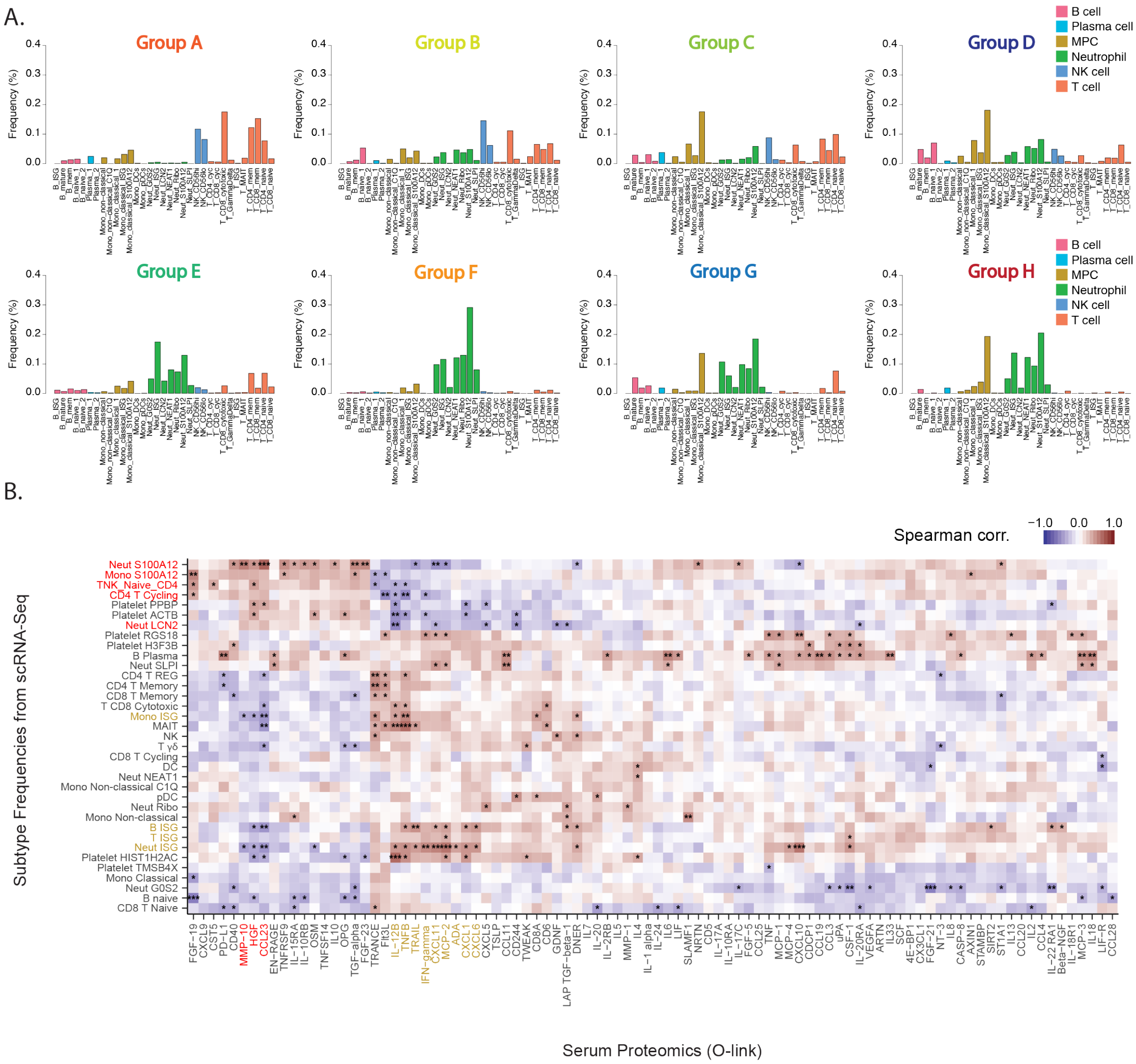


**Supplementary Figure 6: Identifying Immune System Correlates in SARS-CoV-2 positive and negative Patients Data**
**A.** Cell fraction histograms representing bin-wise mean of relative frequency (i.e., cell fraction) of each immune cell subtype for all patients in a given group, colored as described in Fig4A. **B.** Matrix of Spearman correlation coefficients between all subtype frequencies (out of major celltypes e.g. Neut ISG represents % out of all Neutrophils) obtained from scRNA-Seq versus protein analyte abundance in plasma as measured using Olink assay on a patient-by-patient basis excluding healthy controls. Patients for which data were unavailable were excluded from correlation analysis for each comparison. Variables on both axes were ordered via hierarchical clustering. ISG subtypes and protein levels strongly correlated with their frequency highlighted in brown. Subtypes and proteins strongly anti-correlated with ISG+ subtypes highlighted in red. (n=31 for all comparisons) * p<0.05, ** p<0.005, *** p<0.0005.

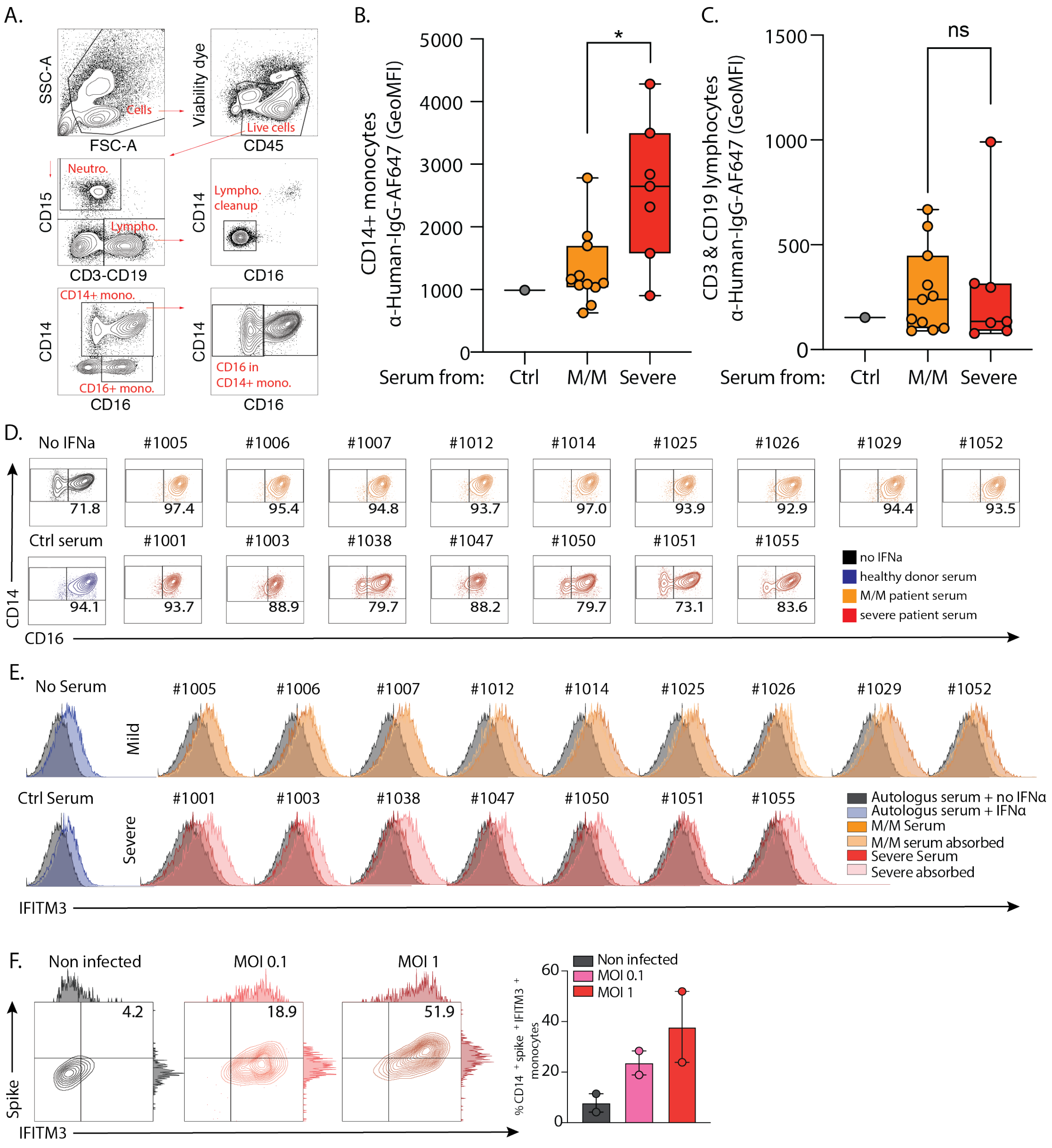


**Supplementary Figure 7. Quantification of Serum Staining and ISG Generation in the Presence of Patient Serum. A.** Gating strategy for PBMCs to identify different subpopulations. **B.** Geometric MFI of serum staining on CD14+ monocytes treated with IFNα, quantifying data in Figure 5A. **C.** Geometric MFI of serum staining of CD3+ and CD19+ lymphocytes, following treatment with IFNα, quantifying data in Figure 5A. **D.** Modulation of intermediate to classical CD14 monocytes transition by mild/moderate (orange) and severe (red) patient serum. Each plot represents a single serum sample. Representative experiment from three independent trials and two different healthy PBMC donors. **E.** Histograms CD3+ CD19+ lymphocytes from healthy donor cultured with IFNa and serum from heathy donor (blue), mild/moderate (orange) and severe (red) SARS-CoV-2 positive patients. Mild/Moderate (light yellow) or Severe (pink) sera were pre-treated with protein G/A before incubation with PBMC. Each plot represents a single serum sample. Representative experiment from two independent trials and two different healthy PBMC donors. **F.** Contour plots and histograms of CD14+ monocytes from healthy donor co-cultured with SARS-COV-2 virus for 48h at 0.1 (pink) and 1 (red) MOI. Differences in B. and C. were calculated using a two-way ANOVA test with multiple comparisons. * p.value < 0.05; ** p.value < 0.01; *** p.value <0.001; **** p.value < 0.0001. ns, non-significant.

## Material and Methods

### Patients, participants, severity score, and clinical data collection

Patients admitted to the Hospital of the University of California with known or presumptive COVID-19 were screened within 3 days of hospitalization. Patients, or a designated surrogate, provided informed consent to participate in the study. This study includes a subset of patient enrolled between April 8 and May 1 in the COMET (COVID-19 Multi-immunophenotyping projects for Effective Therapies; https://www.comet-study.org/) study at UCSF. COMET is a prospective study that aims to describe the relationship between specific immunologic assessments and the clinical courses of COVID-19 in hospitalized patients. Healthy donors (Ctrl) were adults with no prior diagnosis of or recent symptoms consistent with COVID-19. This analysis includes samples from participants who provided informed consent directly, via a surrogate, or otherwise in accordance with protocols approved by the regional ethical research boards and the Declaration of Helsinki. For inpatients, clinical data were abstracted from the electronic medical record into standardized case report forms. We used both a severity score at the time of sampling and at the end of hospitalization (Fig S1A). In both cases, severity assessment was based on three main parameters: level of care, need for mechanical ventilation, and time under mechanical ventilation. Mild/moderate patients are floor/ICU patients who did not require mechanical ventilation during their time of hospitalization and spent no more than 1 day in ICU. Severe patients are patients who required intensive care and mechanical ventilation (typically 5 days or more). Therefore, our cohort is composed of 21 COVID-19 positive patients (11 mild/moderate and 10 severe), 11 COVID-19 negative patient (6 mild/moderate and 5 severe), and 14 Healthy participants. Information on age, sex, type of infection, days of on onset, viral load, and CBC count are listed in Table S1. The study is approved by the Institutional Review board: IRB# 20-30497.

### Isolation of blood cells and processing for scRNA-seq:

ScRNA-seq was performed on fresh whole blood in order to preserve granulocytes. Briefly, peripheral blood was collected into EDTA tubes (BD, catalog no. 366643). Whole blood was prepared by treatment of 500µL of peripheral blood with RBC lysis buffer (Roche, 11-814-389-001) according to manufacturer's procedures. Cells were then counted and 15.000 cells per individual were directly loaded in the Chromium$^{TM}$ Controller for partitioning single cells into nanoliter-scale Gel Bead-In-Emulsions (GEMs) following manufacturer's procedures (10x genomics). Some samples were pooled together (at 15,000 cells/ sample) prior to GEM partitioning. Single Cell 5' reagent kit v5.1 was used for reverse transcription, cDNA amplification and library construction of the gene expression libraries (10x Genomics) following the detailed protocol provided by 10x Genomics. Libraries were sequenced on an Illumina NovaSeq6000 using 28 cycles for R1 and 98 cycles for R2. All samples were encapsulated, and cDNA was generated within 6 hours after blood draw.

### Bulk RNASeq library preparation for Genotyping:

RNA was extracted from aliquots of 250K Peripheral Blood Mononuclear Cells (PBMCs) utilizing the ZYMO Research Quick RNA MagBead kit (R2133) on a Thermofisher KingFisher Flex system following manufacturer's procedures. RNA integrity was inspected with Agilent Fragment Analyzer. Ribosomal and hemoglobin depleted total RNA-sequencing library were created using FastSelect (Qiagen cat#: 335377) and Tecan Universal Plus mRNA-Seq (0520-A01) with adaptations for automation of a Beckmen BioMek FXp system. Libraries were subsequently normalized and pooled for Illumina sequencing using a Labcyte Echo 525 system available at the

Center for Advanced Technology at UCSF. The pooled libraries were sequenced on an Illumina NovaSeq S4 flow cell lane with paired end 150bp reads.

**Computational Processing for Genotyping:**
Sequencing reads were aligned to the human reference genome and Ensembl annotation (GRCh38 genome build, Ensembl annotation version 95) using STAR v2.7.5c (PMID: 23104886) with the following parameters: --outFilterType BySJout --outFilterMismatchNoverLmax 0.04 --outFilterMismatchNmax 999 --alignSJDBoverhangMin 1 --outFilterMultimapNmax 1 --alignIntronMin 20 --alignIntronMax 1000000 --alignMatesGapMax 1000000. Duplicate reads were removed and read groups assigned by individual for variant calling using Picard Tools v2.23.3 (http://broadinstitute.github.io/picard/). Nucleotide variants were identified from the resulting bam files using the Genome Analysis Tool Kit (GATK, v4.0.11.0) following the best practices for RNA-seq variant calling (PMID: 25431634; PMID: 21478889). This include splitting spliced reads, calling variants with HaplotypeCaller (added parameters: --dont-use-soft-clipped-bases -stand-call-conf 20.0), and filtering variants with VariantFiltration (added parameters: -window 35 -cluster 3 --filter-name FS -filter FS > 30.0 --filter-name QD -filter QD < 2.0). Variants were further filtered to include a list of high quality SNP for identification of the subject of origin of individual cells by removing all novel variants, maintaining only biallelic variants with MAF greater than 5%, a mix missing of one individual with a missing variant call at a specific site and requiring a minimum depth of two (parameters: --max-missing 1.0 --min-alleles 2 --max-alleles 2 --remove-indels --snps snp.list.txt --min-meanDP 2 --maf 0.05 --recode --recode-INFO-all –out).

**Data pre-processing of 10x Genomics Chromium scRNA-seq data:**
Sequencer-obtained bcl files were demultiplexed into individual sample the mkfastqs command on the Cellranger 3.0.2 suite of tools (https://support.10xgenomics.com). Feature-barcode matrices were obtained for each sample by aligning the raw fastqs to GRCh38 reference genome (annotated with Ensembl v85) using the Cellranger count. Raw feature-barcode matrices were loaded into Seurat 3.1.5(*2*) and genes with fewer than 3 UMIs were dropped from the analyses. Matrices were further filtered to remove events with greater than 20% percent mitochondrial content, events with greater than 50% ribosomal content, or events with fewer than 100 total genes. The cell cycle state of each cell was assessed using a published set of genes associated with various stages of human mitosis (*3*).

**Inter-sample doublet detection**
Libraries containing samples pooled prior to loading were processed using Freemuxlet (https://github.com/statgen/popscle), the genotype-free version of Demuxlet(*4*), to identify clusters of cells belonging to the same patient via SNP concordance. Briefly, the aligned reads from Cellranger were filtered to retain reads overlapping a high-quality list of SNPs obtained from the 1000 Genomes Consortium (1KG)(*5*). Freemuxlet was run on this filtered bam using the 1KG vcf file as a reference, the input number of samples/pool as a guideline for clustering groups of cells by SNP concordance, and all other default parameters. Cells are classified as singlets arising from a single library, doublets arising from two or more libraries, or as ambiguous cells that cannot be accurately assigned to any existing cluster (due to a lack of sufficient genetic information). Clusters of cells belonging to a unique sample were mapped to patients using their individual Freemuxlet-generated genotype, and ground truth genotypes per patient identified via bulk RNASeq. The pairwise discordance between inferred and ground-truth genotypes was assessed using the bcftools gtcheck command(*6*). Ambiguous, and doublet events were filtered from the major analysis (see platelet-first analysis).

**Data quality control and Normalization:**

The filtered count matrices were normalized, and variance stabilized using negative binomial regression via the scTransform method offered by Seurat(*7*). The effects of mitochondrial content, ribosomal content, and cell cycle state were regressed out of the normalized data to prevent any confounding signal. The normalized matrices were reduced to a lower dimension using Principal Component Analyses (PCA) and the first 30 principal coordinates per sample were subjected to a non-linear dimensionality reduction using Uniform Manifold Approximation and Projection (UMAP). Clusters of cells sharing similar transcriptomic signal were identified using the Louvain algorithm, and clustering resolutions varied between 0. 6 and 1.2 based on the number and variety of cells obtained in the datasets. Clusters were loosely grouped into major cell types (T/NK, B/Plasma, mononuclear phagocytes, Neutrophil, Platelet, and Erythrocytes) using a curated list of 5 genes per cell type (5-gene signature) and the Seurat AddModuleScore function. Briefly, genes in the library are binned into one of 12 bins based on average expression in the dataset. The average expression of the genes in each signature are compared to a background list of randomly selected from the bins and used to generate a score per cell for each signature.

**Intra-sample heterotypic doublet detection**

All libraries were further processed to identify heterotypic doublets arising from the 10X sample loading. Processed, annotated Seurat objects were processed using the DoubletFinder package (*8*). Briefly, the cells from the object are modified to generate artificial duplicates, and true doublets in the dataset are identified based on similarity to the artificial doublets in the modified gene space. The prior doublet rate per library was approximated using the information provided in the 10x knowledgebase (https://kb.10xgenomics.com/hc/en-us/articles/360001378811) and this was corrected to account for homotypic doublets using the per-cluster numbers in each dataset. Heterotypic doublets were removed from the major analysis (see platelet-first analysis).

**Data integration and Batch correction:**

The individual processed objects per library were filtered to remove Erythrocyte contamination. The raw and log-normalized counts per library were then pruned to retain only genes shared by all libraries. Pruned counts matrices were merged into a single Seurat object and the batch (or library) of origin was stored in the metadata of the object. The log-normalized counts were reduced to a lower dimension using PCA and the individual libraries were aligned in the shared PCA space in a batch-aware manner (Each individual library was considered a batch) using the Harmony algorithm (*9*). The resulting Harmony components were used to generate a batch corrected UMAP, and to identify clusters of transcriptionally similar cells. Clusters were broadly labeled based on the 5-gene signature (Fig S1) using a modified, bootstrapped version of the Seurat AddModuleScore to account for the numerous sequencing batches in our dataset. The modified function ran the AddModuleScore on random subsets of the data (subsampling rate = 0.6) 10 times and averaged the score to provide a stable score per signature. A Seurat object was generated for each broad cell type containing clusters scoring highly for that cell type. Each broad cell-type object was subjected to the same harmony analysis to generate batch-aware log normalized counts that were used for visualization and subtype identification. To visualize the effect of harmonizing our single cell data, we identified the library diversity in the neighborhood of every cell on the plot. The neighborhood of a cell is defined as the collection of n nearest neighbors in the UMAP space (where n = sqrt(total cells)), pruned to retain cells lying within the 90th percentile of all calculated neighbor distances. The diversity is the set of all libraries represented within the neighborhood.

**Differential expression tests and cluster marker genes, cluster annotation and volcano plot**
Differential gene expression (DGE) tests were performed on log-normalized gene counts using the Poisson test (with a latent batch variable to account for multiple library preparations) provided by the FindMarkers/FindAllMarkers functions in Seurat. Genes with > 0.35 log-fold changes, an adjusted p value of 0.05 (based on Bonferroni correction), at least 25% expressed in tested groups, were regarded as significantly differentially expressed genes (DEGs). Cluster marker genes were identified by applying the DE tests for upregulated genes between cells in one cluster to all other clusters in the dataset. Top ranked genes (by log-fold changes) from each cluster of interest were extracted for further illustration. The exact number and definition of samples used in the analysis are specified in the legend of Figure 1, 2 and 3 and summarized in Table S1. The neutrophils, mononuclear phagocytic cells, T cells, B cells and platelets subtypes were identified by comparing cluster marker genes with public sources referenced in the text. The R package EnrichR were used to generate volcano plot from differential gene expression using FindMarkers function in Seurat.

**Platelet First scSeq Analysis**
To identify the differential coagulation of platelets, we reintroduced heterotypic doublets to each library and filtered them to extract cells expressing at least 1 UMI of PF4 or PPBP, both platelet-specific marker genes. The raw and log-normalized counts per library were integrated using Harmony and processed as above. Broad cell types were identified using the score generated with the bootstrapped AddModuleScore and the per-sample rate of platelet aggregation with each cell types was inferred to be the fractions of cell counts in this dataset to the fractions of cell counts in the overall analysis. Significance testing was conducted using a non-parametric Kruskal-Wallis test with multiple comparisons.

**Monocle analysis**
Raw counts from the Individual cell-specific were used to create a monocle3 (*10-12*) cell_data_set object, and the batch-corrected PCA and UMAP embeddings were imported directly from the Seurat object. Each cell-specific trajectory was inferred by reverse embedding the UMAP coordinates using the DDRTree method. The root cell states for the trajectory in monocytes and neutrophils were chosen based on literature, and for platelet cell based on the signature list defined in Figure S1. Relative pseudotime was obtained through a linear transformation relative to the cells with the lowest and highest pseudotimes ( *(1-min_pseudotime)/max_pseudotime* ).

**Generation of gene expression scores**
ISG and Degranulation scores were generated by taking the mean of log-normalized expression for a particular set of genes related to the specific pathway or phenotype. The following gene lists were used to generate the scores-ISG: *MT2A, ISG15, LY6E, IFIT1, IFIT2, IFIT3, IFITM1, IFITM3, IFI44L, IFI6, MX1, IFI27*; Degranulation: 486 genes from Neutrophils degranulation GO term #GO:0043312. To visualize the distribution of these scores across cells, we binarized the distribution of the score at the 75th percentile and overlaid on the calculated UMAP coordinates.

**Correlation plots and heatmap visualization**
Correlation coefficients used in variable against variable comparisons were calculated using Spearman's method to avoid assumption of linearity. Significance testing of correlation was performed with the following tests: Spearman's for continuous v. continuous, Kruskal-Wallis or Wilcoxon rank sum test for categorical v. continuous depending on number of categories and Fisher's exact for categorical v. categorical comparisons. Unless otherwise specified, variables on

both axes were hierarchically clustered based on the distance matrix computed from the correlation coefficient.

**Embedding a low-dimensional representation of patients using PhEMD:**
PhEMD was employed to generate a three-dimensional embedding of patients based on their immune cell profiles (*13*). Briefly, PhEMD first generates a reference map of cell subtypes, then uses Earth Mover's Distance (EMD) to compute pairwise dissimilarities between patients (incorporating patient-to-patient differences in cell fractions of each cell subtype as well as intrinsic dissimilarities between subtypes based on the cell subtype reference map), and finally applies a dimensionality reduction technique to the patient-to-patient distance matrix to generate a final embedding of patients. The Seurat implementation of 3D Uniform Manifold Approximation and Projection (UMAP) was used to map the cell-subtype space using the Harmony batch-corrected components as input and a "min.dist" parameter of 0.4, and cell subtypes (i.e., clusters) were defined as described in the "Data quality control and Normalization" section of Methods (*2, 9*). Dissimilarity between each pair of cell subtypes was defined as the distance between the centroids (in UMAP space) of all cells assigned to the two respective subtypes. PHATE was applied to the EMD patient-to-patient distance matrix to generate the final 3D embedding of patients (*14*).

**Luminex Assay for Antibody Titer**
Highly immunogenic linear regions of the SARS-CoV-2 proteome were isolated by ReScan and conjugated to Luminex beads as previously described (*15*). Briefly, high concentration T7 phage stocks displaying immunodominant epitopes of the S, N and ORF3a proteins were propagated and grown to high (>$10^{11}$ PFU/mL) titer then were each conjugated to unique bead IDs according to manufacturer's Antibody Coupling Kit instructions (Luminex). Whole N protein (RayBiotech) beads were conjugated similarly using manufacturer instructions with 5µg of protein per 1 million beads. For other whole protein Luminex-based beads, MagPlex-Avidin Microspheres (Luminex) were coated with either the S protein RBD (residues 328-533) or the trimeric S protein ectodomain (residues 1-1213). All beads were blocked overnight before use and pooled on day of use. 2000-2500 beads per ID were pooled per incubation with patient serum at a final dilution of 1:500 for 1 hour, washed, then stained with an anti-IgG pre-conjugated to phycoerythrin (Thermo Scientific, #12-4998-82) for 30 minutes at 1:2000. Primary incubations were done in PBST supplemented with 2% nonfat milk and secondary incubations were done in PBST. Beads were processed in 96 well format and analyzed on a Luminex LX 200 cytometer. Median Fluorescence Intensity from each set of beads within each bead ID were retrieved directly from the LX200 and log transformed after normalizing to the mean signal across two intra-assay negative controls (glial fibrillary acidic protein (GFAP) and Tubulin phage peptide conjugated beads).

**Luminex Assay for Serum Cytokines**
Soluble proteins were quantified in EDTA anticoagulated plasma using the Luminex® multiplex platform (Luminex, Austin TX) with custom-developed reagents (R&D Systems, Minneapolis, MN), as described in detail ((*16*) or single-plex ELISA (R&D Systems, Minneapolis, MN). Analytes quantified using the Luminex® multiplex platform were read on the MAGPIX® instrument and raw data were analyzed using the xPONENT® software. Analytes quantified using single-plex ELISA were read using optical density. Values outside the lower limit of quantification were assigned a value of 1/3 of the lower limit of the standard curve for analytes quantified by Luminex and 1/2 of the lower limit of the standard curve for analytes quantified by ELISA.

**O-link Assay for Serum Factors**
Circulating proteins were measured in plasma using the multiplexed Proximity Extension Assay (PEA) from Olink Proteomics AB (Uppsala, Sweden). 20 µL each of plasma collected from the COMET patient cohort (21 COVID-19 positive, 13 COVID-19 negative, and 14 healthy individuals) were analyzed using the Olink® Target 96 Inflammation panel, which is a set of 92 inflammation-related protein biomarkers. Plasma for all samples regardless of COVID-19 status were inactivated using a final concentration of 1% (v/v) Triton-X-100 solution over 2 hours. Data from the analyzed protein biomarkers is presented as Normalized Protein eXpression (NPX) values, an arbitrary unit on a log2 scale.

**ELISA Method for Serum IFNα Measure**
IFN- α levels were quantified from serum by an ELISA (catalog numbers 41115 for IFN-α; PBL Assay Science). ELISA was performed according to the manufacturer's instructions with minor modifications. Briefly, an 8- point standard curve was prepared and diluted in sample buffer. Serum was also diluted by adding 80-90 ul serum to 30 ul sample buffer (depending on availability of serum). Samples were prepared in duplicates, whereas standards were prepared in triplicates. Initial incubation was performed for 20 hours at 4C. Antibody was added and incubated at 4C overnight. HRP was added and incubated 1hr at room temperature, TMB substrate was added for 30min and incubated in the dark at room temp, stop solution was added and samples were read using a SpectraMax M2 Microplate Reader (Molecular Devices) at 450nm. For analysis, a 4-parameter logistic fit was applied to OD values of the standards after background subtraction. Samples with ODs below blank samples were considered as 0 pg/ml IFN- α.

**PBMC co-culture experiment with patient serum and flow cytometry analysis**
PBMCs were isolated from EDTA-anticoagulated whole blood from healthy donors using Polymorphprep (Alere Technologies), and resuspended in culture medium (RPMI 1640 + 10% FBS). For detection of neutralization of interferon stimulation, autologous serum or clinical study participant sera (10 µl) were plated with IFNα (Stemcell IFN alpha-2A; final concentration of 1 pg/ml) in a total volume of 100µl before addition of 4×105 PBMCs. After incubation for 24 hours, PBMCs were assayed for IFNα-induced IFITM3 upregulation and CD14/CD16 levels and fractions by flow cytometry. After surface staining and addition of fixable live/dead violet dye (ThermoFisher; #L34955), intracellular detection of IFITM3 was done using the eBioscience Foxp3 / Transcription Factor Staining Buffer Set (ThermoFisher; #00-5523-00) and following the manufacturer's instructions. For autoantibody assays, PBMCs were cultured with media or 1-100 pg/ml IFNα for 38-46 hours. Samples were harvested and unconjugated AffiniPure Fab Fragment Goat anti-human IgG (H+L) (Jackson Immunoresearch; #109-007-003) and Human TruStain FcX block (BioLegend; #422302) were used to block pre-bound antibodies and Fc receptors. After washing with fluorescence-activated cell sorting (FACS) buffer (2% fetal bovine serum, 1 mM EDTA, PBS), PBMCs were then stained for surface markers 30 min on ice. After staining incubation, cells were washed 3x times with FACS buffer (1500 rpm, 5 min, 4°C) and incubated with 5µl autologous or clinical study participant sera for 90 min on ice. After washing the cells with FACS buffer, cell-bound antibodies were detected using an AffiniPure Donkey anti-human IgG-Alexa Fluor 647 antibody (Jackson Immunoresearch; #709-605-149), which was incubated with the cells for 30 min on ice. Cells were washed again and resuspended in 1 µg/ml DAPI solution for live/dead discrimination. The following antibodies were used for flow cytometric analysis: anti-human CD3-BB700 (clone SK7; BD Biosciences; #566575), anti-human CD14-BV711 (clone MSE2; BioLegend; #301838), anti-human CD15-BV786 (clone W6D3; BD Biosciences; #741013), anti-human CD16-BV605 (clone 3G8; BioLegend; #302040), anti-human CD19-BB700 (clone SJ25C1; BD Biosciences;

566396), anti-human CD45-APCeFluor780 (clone HI30; ThermoFisher; 47-0459-42), anti-human IFITM3-AlexaFluor 647 (clone EPR5242; Abcam; ab198573).

**Bead ELISA:**
$10^7$ 5um Sulfate latex polystyrene beads (Thermo Fisher) were resuspended in 1ml of PBS to which 1ug of proteins (BSA, huIFN$\alpha$ (Stemcell IFN alpha-2A) were added to bind by passive absorption over 1 hr on ice. Beads were washed 1x in PBS and blocked with 1ml of blocking buffer (PBS containing 1mM EDTA and 2%FCS) for one hour. Beads were spun and resuspended in 1ml of blocking buffer and 10ul ($10^5$ beads) were moved to individual tubes to which 5ul of sera was added followed by incubation for 1hr on ice). These were washed, resuspended in 50ul of blocking buffer containing Goat anti-human IgG-Alexa Fluor 647 (Jackson Immunoresearch) and incubated for 1hr on ice followed by a final wash and analysis by flow cytometry.

**SARS-CoV-2 detection by PCR**
PCR testing for SARS-CoV-2 was carried out on respiratory specimens mixed 1:1 in DNA/RNA Shield (Zymo Inc) using an in-house Clinical Laboratory Improvement Amendments (CLIA) validated assay at the UCSF Clinical Microbiology Laboratory. PCR primers targeted the SARS-CoV-2 envelope (E) and RNA-dependent RNA polymerase (RdRp) genes plus human RNAse P as a positive control.

**SARS-CoV-2 infection of PMBC**
SARS-CoV-2 isolate USA-WA1/2020 was provided by Dr. Melanie Ott and propagated in Vero E6 (ATCC CRL-1586) cells in Dulbecco's Modified Eagle Medium (UCSF Cell Culture Facility) supplemented with 10% FBS. Vero E6 cells were infected with the SARS-CoV-2 virus for 72h at 37C and 5% CO2. The supernatant was collected and viral titer was quantified using a plaque assay in Vero E6 cells. All work was done under BSL3 conditions. PBMC were infected with SARS-CoV-2 virus at a multiplicity of infection (MOI) 0.1 or 1 for 72 hours. Cells were then harvested and stained with fixable Live/Dead Zombie NIR (Biolegend) in PBS followed by fixation with 4% paraformaldehyde for 1 hour. Intracellular staining to Spike (SARS-CoV-2 Spike S1 Antibody, Rabbit mAb (SinoBiological) and IFITM3 was subsequently performed using eBioscience Foxp3/Transcription Factor Staining Buffer Set (Thermo Fisher Scientific) followed by surface antigen staining.

**Statistical Analysis and Data visualization**
Statistical analyses were performed using GraphPad prism or the R software package. Null hypotheses between two groups were tested using the non-parametric Mann-Whitney test to account for non-normal distribution of the data. Likewise, for multiple groups, comparisons were made by two-way ANOVA or non-parametric Kruskal–Wallis test followed by multiple comparisons. The specific statistical tests and their resultant significance levels are also noted in each figure legend. The R packages Seurat, ggplot2 (version 3.1.0) (Wickham, 2016) GraphPad Prism and Adobe Illustrator were used to generate figures.

**Data Resources and Code Sharing:**
Raw Gene expression matrices will be available on GEO at the time of publication. Scripts used to process all data will be shared on Github along with relevant clinical information for each patient.

## References

1. D. Bongiovanni *et al.*, Transcriptome Analysis of Reticulated Platelets Reveals a Prothrombotic Profile. *Thrombosis and haemostasis* **119**, 1795-1806 (2019).
2. T. Stuart *et al.*, Comprehensive Integration of Single-Cell Data. *Cell* **177**, 1888-1902 e1821 (2019).
3. D. Dominguez *et al.*, A high-resolution transcriptome map of cell cycle reveals novel connections between periodic genes and cancer. *Cell research* **26**, 946-962 (2016).
4. H. M. Kang *et al.*, Multiplexed droplet single-cell RNA-sequencing using natural genetic variation. *Nat Biotechnol* **36**, 89-94 (2018).
5. C. Genomes Project *et al.*, A global reference for human genetic variation. *Nature* **526**, 68-74 (2015).
6. H. Li, A statistical framework for SNP calling, mutation discovery, association mapping and population genetical parameter estimation from sequencing data. *Bioinformatics* **27**, 2987-2993 (2011).
7. C. Hafemeister, R. Satija, Normalization and variance stabilization of single-cell RNA-seq data using regularized negative binomial regression. *Genome biology* **20**, 296 (2019).
8. C. S. McGinnis, L. M. Murrow, Z. J. Gartner, DoubletFinder: Doublet Detection in Single-Cell RNA Sequencing Data Using Artificial Nearest Neighbors. *Cell Syst* **8**, 329-337 e324 (2019).
9. I. Korsunsky *et al.*, Fast, sensitive and accurate integration of single-cell data with Harmony. *Nat Methods* **16**, 1289-1296 (2019).
10. X. Qiu *et al.*, Reversed graph embedding resolves complex single-cell trajectories. *Nat Methods* **14**, 979-982 (2017).
11. X. Qiu *et al.*, Single-cell mRNA quantification and differential analysis with Census. *Nat Methods* **14**, 309-315 (2017).
12. C. Trapnell *et al.*, The dynamics and regulators of cell fate decisions are revealed by pseudotemporal ordering of single cells. *Nat Biotechnol* **32**, 381-386 (2014).
13. W. S. Chen *et al.*, Uncovering axes of variation among single-cell cancer specimens. *Nat Methods* **17**, 302-310 (2020).
14. K. R. Moon *et al.*, Visualizing structure and transitions in high-dimensional biological data. *Nat Biotechnol* **37**, 1482-1492 (2019).
15. C. R. Zamecnik *et al.*, ReScan, a Multiplex Diagnostic Pipeline, Pans Human Sera for SARS-CoV-2 Antigens. *medRxiv*, 2020.2005.2011.20092528 (2020).
16. A. Leligdowicz *et al.*, Validation of two multiplex platforms to quantify circulating markers of inflammation and endothelial injury in severe infection. *PLoS One* **12**, e0175130 (2017).

**Supplementary Table of Authors from the UCSF COMET Consortium**


| Name | Institution |
|---|---|
| Cathy Cai | Department of Pathology and ImmunoX, UCSF, San Francisco, California, USA. |
| Jenny Zhan | Department of Pathology and ImmunoX, UCSF, San Francisco, California, USA. |
| Bushra Samad | Department of Pathology and ImmunoX, UCSF San Francisco, California, USA. |
| Kwok W. Im | Department of Pathology and ImmunoX, UCSF San Francisco, California, USA. |
| Nina K. Serwas | Department of Pathology and ImmunoX, UCSF San Francisco, California, USA. |
| Suzanna Chak | Division of Pulmonary and Critical Care Medicine, Department of Medicine, UCSF, San Francisco, California, USA. |
| Rajani Ghale | Division of Pulmonary and Critical Care Medicine, Department of Medicine, UCSF, San Francisco, California, USA. |
| Jeremy Giberson | Division of Pulmonary and Critical Care Medicine, Department of Medicine, Zuckerberg San Francisco General Hospital and Trauma Center, UCSF, San Francisco, California, USA. |
| Ana Gonzalez | Division of Pulmonary and Critical Care Medicine, Department of Medicine, Zuckerberg San Francisco General Hospital and Trauma Center, UCSF, San Francisco, California, USA. |
| Alejandra Jauregui | Division of Pulmonary and Critical Care Medicine, Department of Medicine, UCSF, San Francisco, California, USA. |
| Deanna Lee | Division of Pulmonary and Critical Care Medicine, Department of Medicine, Zuckerberg San Francisco General Hospital and Trauma Center, Cardiovascular Research Institute, UCSF, San Francisco, CA, USA. |
| Viet Nguyen | Division of Pulmonary and Critical Care Medicine, Department of Medicine, Zuckerberg San Francisco General Hospital and Trauma Center, Cardiovascular Research Institute, UCSF, San Francisco, CA, USA. |
| Kimberly Yee | Division of Pulmonary and Critical Care Medicine, Department of Medicine, University of California San Francisco, Cardiovascular Research Institute, UCSF, San Francisco, CA, USA. |
| Yumiko Abe-Jones | Division of Hospital Medicine, UCSF, San Francisco, California, USA. |
| Logan Pierce | Division of Hospital Medicine, UCSF, San Francisco, California, USA. |
| Priya Prasad | Division of Hospital Medicine, UCSF, San Francisco, California, USA. |
| Pratik Sinha | Division of Pulmonary and Critical Care Medicine, Department of Medicine, UCSF, San Francisco, California, USA. |
| Alexander Beagle | Department of Medicine, UCSF San Francisco, California, USA |
| Tasha Lea | Department of Pathology, UCSF San Francisco, California, USA. |
| Armond Esmalii | Division of Hospital Medicine, University of California, San Francisco, CA, USA. |
| Austin Sigman | Division of Pulmonary and Critical Care Medicine, Department of Medicine, University of California San Francisco, San Francisco, California, USA. |
| Gabriel M Ortiz | Department of Medicine, Zuckerberg San Francisco General Hospital and Trauma Center, University of California San Francisco |
| Kattie Raffel | Division of Hospital Medicine, University of California, San Francisco, CA, USA. |

| | |
|---|---|
| Chayse Jones | Division of Pulmonary and Critical Care Medicine, Department of Medicine, University of California San Francisco, San Francisco, California, USA. |
| Kathleen Liu | Division of Nephrology, Department of Medicine, University of California at San Francisco School of Medicine, San Francisco, CA, United States<br>Division of Critical Care Medicine, Department of Anesthesia, University of California at San Francisco School of Medicine, San Francisco, CA, United States. |
| Walter Eckalbar | Division of Pulmonary and Critical Care Medicine, Department of Medicine, Cardiovascular Research Institute and CoLabs, UCSF, San Francisco, CA, USA. |
